# *Salmonella* Degrades the Host Glycocalyx Leading to Altered Infection and Glycan Remodeling

**DOI:** 10.1038/srep29525

**Published:** 2016-07-08

**Authors:** Narine Arabyan, Dayoung Park, Soraya Foutouhi, Allison M. Weis, Bihua C. Huang, Cynthia C. Williams, Prerak Desai, Jigna Shah, Richard Jeannotte, Nguyet Kong, Carlito B. Lebrilla, Bart C. Weimer

**Affiliations:** 1 Department of Population Health and Reproduction, School of Veterinary Medicine, University of California, Davis, CA 95616, USA; 2 Department of Chemistry, University of California, Davis, CA 95616, USA; 3 Universidad de Tarapacá, Avenida General Velásquez N°1775, Arica, Chile; 4 Department of Biochemistry and Molecular Medicine, School of Medicine, University of California, Davis, CA 95616, USA

## Abstract

Complex glycans cover the gut epithelial surface to protect the cell from the environment. Invasive pathogens must breach the glycan layer before initiating infection. While glycan degradation is crucial for infection, this process is inadequately understood. *Salmonella* contains 47 glycosyl hydrolases (GHs) that may degrade the glycan. We hypothesized that keystone genes from the entire GH complement of *Salmonella* are required to degrade glycans to change infection. This study determined that GHs recognize the terminal monosaccharides (*N*-acetylneuraminic acid (Neu5Ac), galactose, mannose, and fucose) and significantly (p < 0.05) alter infection. During infection, *Salmonella* used its two GHs sialidase *nanH* and amylase *malS* for internalization by targeting different glycan structures. The host glycans were altered during *Salmonella* association via the induction of *N*-glycan biosynthesis pathways leading to modification of host glycans by increasing fucosylation and mannose content, while decreasing sialylation. Gene expression analysis indicated that the host cell responded by regulating more than 50 genes resulting in remodeled glycans in response to *Salmonella* treatment. This study established the glycan structures on colonic epithelial cells, determined that *Salmonella* required two keystone GHs for internalization, and left remodeled host glycans as a result of infection. These data indicate that microbial GHs are undiscovered virulence factors.

Epithelial cells in the human gastrointestinal tract (GIT) are covered with at least two glycan layers, composed of multiple layers of glycoproteins (mucin) and complex oligosaccharides (glycocalyx) that protect cells from the local environment and infection^1^. Mucin is the most distal layer of glycoproteins in the GIT lumen and is directly exposed to the luminal microbiome. The glycocalyx layer is adjacent the epithelial membrane that is composed of trans-membrane glycoproteins and glycolipids and are components of membrane lipid rafts that extend from the membrane, which are specific bacterial and viral receptors used for microbial invasion resulting in transduction of extracellular signals into the cell^2^,^3^,^4^,^5^. Glycans represent the first and crucial interface of the cell surface through which microbes interact with the host immune system mediating recognition and communication processes; thus, controlling immunological recognition, cell-cell adhesion, and pathogen binding. Glycans are mixture of structures that include short chains of saccharides, with simple structures to highly branched complex oligosaccharides that are complicated with an array of specific linkages between the monosaccharide residues leading to a large diversity in arrangements during their synthesis to form higher order chemical structures^1^. In the case of bacterial pathogens, this layer provides a barrier to physically exclude microorganisms from gaining access to the epithelial membrane and the associated receptors used for infection and have been suggested to be used for co-evolution with commensal bacteria^2^.

Bacterial interaction with the epithelial surface via the glycan has been recognized for many years with the use of lectins to identify microbial activity^6^. Beyond system level interactions specific interactions with via fucose and sialic acid have been implicated to regulate commensal interactions and provide sugar sources in complex communities where some members may cleave the sugar for use by other community members^6^,^7^,^8^,^9^,^10^. The specific linkages (i.e. α-1,2/3/4 and α-2,3/6 linkages), in addition to these specific sugars are also implicated in controlling the microbial interaction^11^. The breath of the microbes that interact with mucin and the underlying glycocalyx interactions is large and has been suggested to be a primary underpinning of co-evolution of an individual and their microbiome^11^,^12^. In fact, recognition of the glycan as the primary interacting surface has also led to detailing the very large and complex carbohydrate digestion enzymes in bacteria that are assembled in CAZymes^13^. Use of the host glycan to provide nutrients that regulate bacterial infection and virulence is increasingly recognized as an important characteristic for individual microbes to penetrate the mucin layers and subsequently gain access to the cell membrane for association and invasion to progress into the disease state^14^,^15^.

*Salmonella*, and other invasive enteric pathogens, developed mechanisms to breach the protective glycan layers of host cells, which permits access to the host membrane receptors, that results in invasive intracellular infection. The diversity and complexity of the entire epithelial glycan barrier mandates that a breadth of enzymatic activities is required to degrade these diverse glycans to gain access of the cell membrane. Degradation of these compounds relies in part on glycosyl hydrolases (GHs). GHs are diverse, widely distributed in bacteria, yet poorly characterized enzymes, which hinder determining their specific role during infection dynamics^16^,^17^. To date there are no studies that define the specific role of the diverse GHs found in *Salmonella* during infection^16^,^17^. In this study, we hypothesized that only a portion of the 21 different GH families that encompass 49 total carbohydrate-active enzymes in *Salmonella* may degrade glycans to gain access of the host membrane and the microbe receptors used for invasion. Eilam *et al*. demonstrated that microbe’s glycan degradation potential is associated with gut pathogens^18^. It was hypothesized in this study that unique and specific GHs in *Salmonella* are required for invasive infection.

Here, we conducted a detailed study of how *Salmonella* degrades the glycocalyx layer of human colonic epithelial cells (Caco-2) during *in vitro* infection and led to host glycan remodeling. We established that two GHs, *ΔnanH* and *ΔmalS*, decrease invasion to non-invasive levels comparable to *ΔinvA*, suggesting that these GHs may be new virulence factors. The *N*-glycome of Caco-2 cells during infection was profiled and showed how *Salmonella* used its glycan-degrading enzymes, *nanH* and *malS*, to degrade the glycocalyx layer. More surprisingly, we identified that the host cell responded to microbial glycan degradation by modifying its own glycans leading to decreased sialylation; however, increased fucosylation, higher-mannose, and more hybrid glycans. This alteration in host glycans was due to induction of *N*-glycan biosynthesis pathways during *Salmonella* association. This study demonstrates that a complex molecular interplay between epithelial cells and pathogens result in altered association, but infection proceeds if bacteria have the appropriate GH compliment to overcome the dynamic changes in the glycan.

## Results

### Glycan Degrading Enzymes Alter Host Membrane Access during Invasion of *Salmonella*

**Figure 1:**
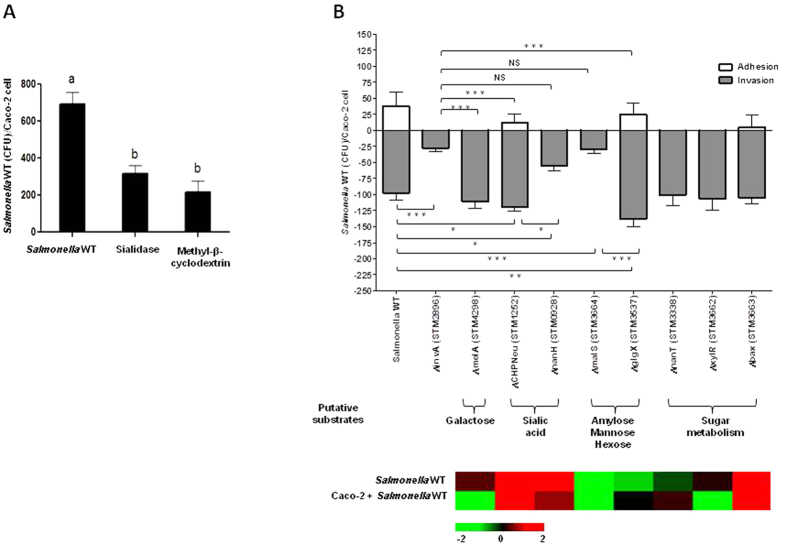
Modulation of carbohydrate-degrading enzymes alter access during invasion of *Salmonella* WT. (**A**) Neu5Ac digestion. Methyl-β-cyclodextrin (control) disrupts lipid rafts. Sialidase treatment led to reduction in *Salmonella* association. Least Squares Means Differences (LSD) was used for statistical analysis. Levels not connected with the same letter are significantly different, p < 0.05. (**B**) *Salmonella* WT knockout strains characterized for the alteration in adhesion and invasion (A/I) using differentiated Caco-2 cells 60 minutes post-infection. White bars represent the CFU of *Salmonella* WT that adhered per Caco-2 cell. The gray bars represent the CFU of *Salmonella* WT that invaded per Caco-2 cell. This was done in combination with transcriptional profiling (bottom panel) of *Salmonella* WT during infection of Caco-2 cells to gain insights about differentially expressed carbohydrate-degrading genes. *Salmonella* WT genes displaying changes in gene expression levels during infection of Caco-2 cells. Colors indicate the expression of each gene induced (red) and repressed (green). LSD was used for statistical analysis. Error bars indicate SEM between 3 biological replications, *p < 0.05, **p < 0.001, ***p < 0.0001, Not Significant (NS). The statistics shown at the top indicates the statistical relevance in invasion levels of *ΔinvA* compared to the mutant strains.

To determine if glycans are involved during infection, we first investigated if the removal of Neu5Ac (the outermost monosaccharide on most *N*-glycans of animals) from Caco-2 cells affected *Salmonella* attachment. Caco-2 cells were exposed to sialidase digestion to remove terminal Neu5Ac. As a control, Caco-2 cells were also subjected to methyl-β-cyclodextrin (MβCD) treatment since MβCD inhibits caveolae-dependent endocytosis via disruption of lipid rafts^19^. Depletion of cell surface Neu5Ac led to a significant (p < 0.05) reduction in *Salmonella* association (Fig. 1A), further implicating the importance of Neu5Ac in the adherence process. To determine if genes related to metabolism of sugars released from glycan are involved during infection, *nanT* (STM3338; sialic acid transporter), *xylR* (STM3662; xylose operon regulatory protein), and *bax* (STM3663; hypothetical protein similar to ATP-binding protein) were deleted using homologous recombination^20^, characterized in their capacity to infect, and for alteration in invasion *in vitro* with Caco-2 cells (Fig. 1B). Deletion of these genes did not significantly change association compared to *Salmonella enterica* subsp. *enterica* serovar Typhimurium strain LT2, suggesting that genes related to metabolism of sugars are not involved in additional invasion.

Deletion of each gene was verified using whole genome sequencing of the wild type and each deletion mutant (Supplementary Figure S1B and C). Reference based assembly and *de novo* assembly showed that the genomes were isogenic, that only the gene of interest was specifically replaced with the chloramphenicol resistance gene, and that the genome-to-genome distance was not significantly different (p > 0.001). Taken together, these data indicate that the gene deletions were adequate to delineate the multi-gene effect demonstrated in this work to digest complex glycans to gain access to the host membrane.

To further test the hypothesis if glycan-degrading GHs alter host membrane access during infection, we analyzed the gene expression of the annotated GHs of *Salmonella* Typhimurium LT2 during *in vitro* infection of Caco-2 cells (Supplementary Table S1). Knowing the most common terminal residues on glycans (i.e. Neu5Ac, galactose, and mannose), GHs that can cleave the most common residues and linkages, along with gene expression analysis, we identified that sialidases (STM0928 and STM1252), galactosidase (STM4298), and amylases (STM3664 and STM3537) likely play an important role during infection. To determine if these GHs are importand for infection, these GHs in *Salmonella* Typhimurium LT2 were also deleted (Supplementary Figure S1A) and used to characterize their ability to infect and alter invasion *in vitro* using differentiated (i.e. polarized) colonic epithelial cells (Caco-2 cells) (Fig. 1B). These enzymes targeted different glycans based on their terminal sugar specificity, as seen with the differences in the infectivity of each bacterial mutant (Fig. 1B), further implicating terminal sugar digestion could be important during infection so that the T3SS can gain access to the membrane to initiate injection of effector molecules. α-Galactosidase (STM4298; *melA*) recognizes terminal α-galactose molecules of glycans. Deletion of *melA* led to a similar invasion phenotype as the WT.

*Salmonella* LT2 contains two sialidases in its genome: *nanH* (STM0928) and *CHPNeu* (STM1252; ***C***onserved ***H***ydrolase ***P***utative ***Neu***raminidase), both of which were deleted from the genome in this study. Genetic comparison of these genes found that *nanH* has homology with genes from bacteria and parasites (Supplementary Figure S2A). Gene homology for *CHPNeu* was broadly distributed among bacteria from the gut and environment (Supplementary Figure S2B and S2C). In *Salmonella* there is very little DNA sequence homology between *nanH* and *CHPNeu*; however, the domain structures were conserved, which allowed identification of function between organisms to provide molecular markers to define two different neuraminidases in *Salmonella* that are enzymatically, and likely play different roles different during infection (Supplementary Figure S3). Nitrogenases among microbial genomes also display wide sequence diversity, but retain conservation of enzymatically important domains^21^. Deletion of *nanH* significantly decreased invasion (p = 0.0059). The *ΔCHPNeu* mutant significantly increased invasion (p = 0.01).

The amylase genes (*malS* and *glgX*) are also divergent in their sequence, but both contain the required domains for amylase activity (Supplementary Figure S4). Deletion of *malS* (STM3664; α-amylase) significantly decreased *Salmonella* invasion (p < 0.0001), resulting in the same level of invasion as *ΔinvA* (STM2896; needle complex export protein for T3SS), which is considered non-pathogenic and deficient for adhesion and invasion *in vivo*^22^. Surprisingly, deletion of *glgX* (STM3537; glycogen debranching enzyme), which is an amylase-like enzyme with broader hexose specificity, led to a significant increase in invasion (p = 0.0002). Perhaps the deletion of *CHPNeu* and *glgX* affected other genes involved in virulence, such as the *nanH* and *malS*. The invasion levels of *nanH* and *malS* mutants were not significantly different (p > 0.05) compared to those found for *ΔinvA*; hence, *nanH* and *malS* may represent new virulence genes in *Salmonella* infections. These observations led to the hypothesis that *Salmonella* targets specific glycan structures during infection that enable the T3SS needle complex access to the membrane since it is not long enough to penetrate the glycan structures on epithelial cells.

### *Salmonella* Infection Led to Alteration in Host *N*-Glycome

**Figure 2:**
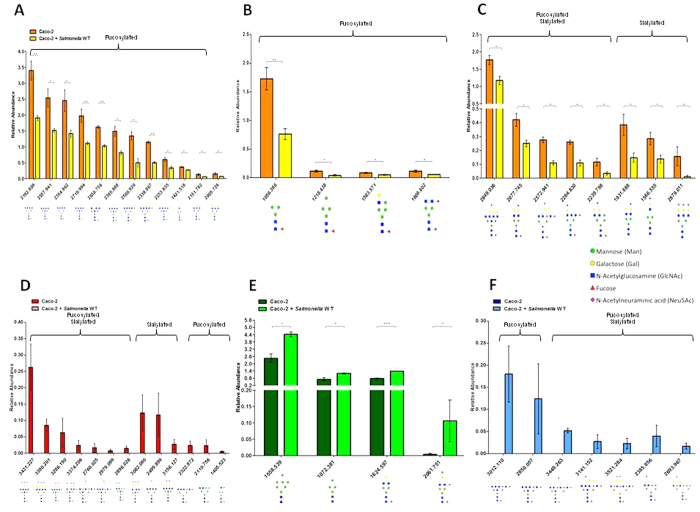
Host glycome is substantially altered during infection with *Salmonella* WT within 60 minutes. The bars represent the relative abundance levels of each significant glycan during infection with *Salmonella*. (**A**) Decrease in complex-fucosylated glycans; (**B**) Decrease in high-mannose and hybrid glycans; (**C**) Decrease in sialylated glycans; (**D**) Disappearance of sialylated glycans; (**E**) Accumulation of high-mannose and complex glycans; (**F**) New complex-fucosylated and sialylated glycans. Error bars indicate SEM between 3 biological replications, *p < 0.05, **p < 0.001, ***p < 0.0001.

We initiated the glycan definition by determining the glycan structure and composition of uninfected Caco-2 cells to define the *N*-glycome baseline to allow comparison of the changes made by *Salmonella* during infection. This approach is of particular interest because it extends the specificity beyond binding via lectin-like activities and can be directly linked to enzyme activity and genes in *Salmonella* that are required to infect. The changes in host N-linked glycan profile are of particular interest as they comprise approximately 90% of the glycans present in eukaryotic cells and modification in these types of glycans has been linked to multiple diseases^23^, but no specific structure has been directly linked to infection even though specific sugars (i.e. sialic acid and fucose) are known to impact the microbiome and association with the host. Uninfected Caco-2 cells contained 191 distinct glycan structures (Supplementary Figure S5A, Supplementary Table S2). The Caco-2 *N*-glycans were rich in mannose, galactose, *N*-acetylglucosamine, fucose, and *N*-acetylneuraminic acid (Neu5Ac). The relative abundance of specific structures changed significantly (p < 0.05) during *Salmonella* infection (Supplementary Figure S5B, Supplementary Table S2) when compared to uninfected cells (Supplementary Figure S5A, Supplementary Table S2). Within 60 minutes of *Salmonella* infection, the host glycome contained substantially different structures compared to uninfected Caco-2 cells (Supplementary Figure S5B) that led to 185 total glycan structures. During infection 13 glycans completely disappeared (Fig. 2D, Supplementary Table S2) and seven glycans were appeared on the host surface (Fig. 2F, Supplementary Table S2). There were also some glycans with intermediate abundance changes with decreases (Fig. 2A–C, Supplementary Table S2) while others increased (Fig. 2E, Supplementary Table S2), yet other glycans were similar to uninfected Caco-2 cells (Supplementary Table S2). Interestingly, the glycans resulting from infection were composed of high-mannose-containing structures and complex glycans that were polyfucosylated with the terminal branches containing up to five fucose molecules, while the majority of the glycans that disappeared were polysialylated.

### Host Cells Modify Their Own Glycan during Microbial Glycan Degradation

**Figure 3:**
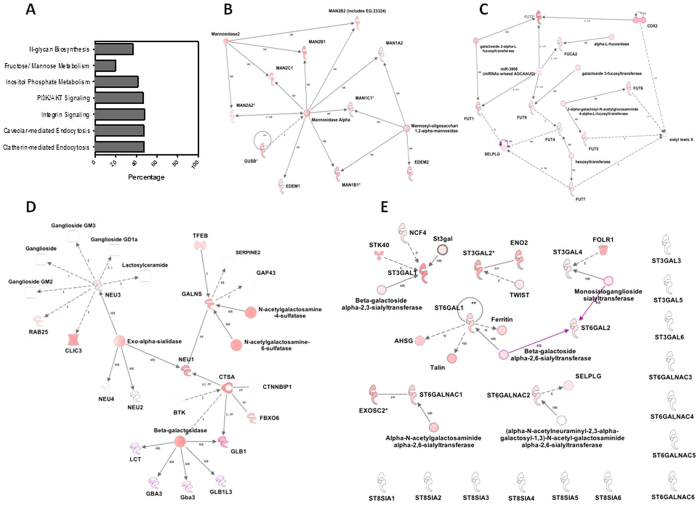
Host responds to microbial glycan degradation by modifying its own glycan. (**A**) Analysis of host pathways involved during glycan degradation of Caco-2 cells following microbial association. Canonical pathways whose biological functions were influenced based on gene expression changes are shown (Fishers exact test). Upregulated molecules in each pathway are represented as a percentage of the total canonical pathway membership. (**B**–**E**) Networks display interactions between genes involved in mannose, fucose and Neu5Ac ([D] sialidases and [E] sialyltransferases) metabolism, respectively, in Caco-2 cells treated for 60 minutes with *Salmonella* LT2. ST3GAL family sialyltransferases catalyzed the addition of Neu5Ac to a terminal galactose of glycoconjugates in an α-2,3 linkage. The sialyltransferases in ST6GAL family transferred alpha-2,6 linking Neu5Ac to galactose residues of *N*-glycans. The ST6GALNAc family sialyltransferases added Neu5Ac to terminal *N*-acetylgalactosamine residues of glycoproteins and glycolipids, in an α-2,6 linkage. Lastly, the ST8Sia family catalyzes the transfer of Neu5Ac in an alpha-2,8 linkage to other Neu5Ac residue present in *N*- or *O*-glycans of Neural cells. Caco-2 up-regulation of enzymes in both gene networks is indicative of microbial induced changes in host glycan biosynthesis. Gene induction is represented as a log ratio (q < 0.05) and displayed in shades of red.

Using gene expression and canonical pathway analysis we examined the host response during infection and glycan degradation. Significantly differentially (q < 0.05) regulated genes following *Salmonella* infection were marked by the induction of genes directly involved in glycan biosynthesis (Fig. 3A, Supplementary Table S3). All mannosidases and mannosyltransferases were all significantly induced (q < 0.05) (Fig. 3B) that was concomitantly observed in the accumulation of mannose in the glycan structures using mass spectrometry described above. The fucosidase, FUCA2, and all seven fucosyltransferases were significantly induced (q < 0.05) (Fig. 3C) that, again was concomitantly observed in the increase in fucose at the terminal residue in the cellular glycan. The *Salmonella* genome does not contain fucosidases, so it is reasonable that these glycans accumulate via host production and modification.

**Figure 4:**
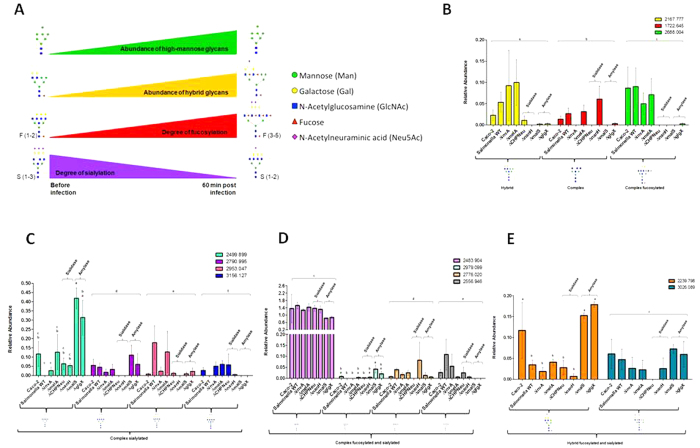
Signature of glycan profiles and how each glycan-degrading enzyme modulates the glycans during infection. (**A**) Signature of glycan profiles during infection. There is an increase in abundances of high-mannose and hybrid glycans. Also the glycans are switched from being highly sialylated to highly fucosylated (the degree of fucosylation increases and the degree of sialylation decreases); (**B**–**E**) Glycans that are highly regulated by *Salmonella* glycan-degrading enzymes during infection. The comparisons between *CHPNeu* and *nanH* (Sialidases) and *malS* and *glgX* (Amylases) are shown. LSD was used for statistical analysis. Error bars indicate SEM between 3 biological replications. Levels not connected with the same letter are significantly different. Statistical analysis was done within each structure.

The overall Neu5Ac content of the glycan was a result of removal by *Salmonella.* We observed induction of NEU1, NEU2, NEU3, and NEU4 in the host led us to presume that these genes were collectively responsible for additional modification of the glycan. The four exo-α-sialidases in the host, each of which has a different substrate specificity, were regulated independently with two induced and two unregulated, had median expression levels. NEU2 and NEU4 were not regulated during infection (Fig. 3D). NEU1, which is commonly located at the lysosome and NEU3, found on cell surfaces, were induced during infection (Fig. 3D). The two sialidases in *Salmonella* were induced during infection, suggesting that Neu5Ac would be released. The glycans were depleted in Neu5Ac during infection, which may indicate the cooperative activity of the host and the pathogen resulted in a reduction of the degree of sialylation from three to one (Fig. 4).

### Glycan Degrading Enzymes Modulated Glycans during Infection

To establish the definitive role of the infection-associated glycan-degrading enzymes, we tested the glycan degradation capacity of *Salmonella* enzymes determined during *in vitro* infection of Caco-2 cells with WT *Salmonella*, *ΔmelA*, *ΔCHPNeu*, *ΔnanH*, *ΔmalS*, and *ΔglgX*. It was shown that GHs impacting invasive infection would be the same or less than *ΔinvA* (Fig. 1B). Bacterial degradation of the Caco-2 glycans is required for invasion. Glycan profiles of the host cell after infection showed similar composition, all of which contained increased mannose content. During infection, the degrees of fucosylation and sialylation also shifted to have higher fucosylation and less sialylation (Fig. 4A). The host cell glycan composition was not expected to be altered during infection with *ΔinvA* since it contained all the glycan-degrading enzymes found in the WT. However, 11 different structures were observed compared to the WT, while the fucose and Neu5Ac content remained unchanged. The host glycan composition remained unchanged when infected with *ΔmelA* with six glycan differences compared to WT, which may explain the similar invasion phenotype as compared to the WT (Fig. 1B). Further examination of the glycans led to identification of specific structures that uniquely linked to *Salmonella* GHs, specifically sialidases and amylases, and regulated infection (Fig. 4B–E). While sialidases are required to initiate glycan degradation we also observed that other glycan-degrading enzymes aid to complete the degradation of these glycans to invade. Glycans m/z = 2499.899 (Hex_6_HexNAc_6_Neu5Ac_1_), 2790.995 (Hex_6_HexNAc_6_Neu5Ac_2_), 3156.127 (Hex_7_HexNAc_7_Neu5Ac_2_), 2979.099 (Hex_5_HexNAc_7_Fuc_3_Neu5Ac_1_), 2239.798 (Hex_6_HexNAc_4_Fuc_1_Neu5Ac_1_), and 3026.089 (Hex_6_HexNAc_5_Fuc_3_Neu5Ac_2_) are sialylated glycans that were degraded during the infection with WT (Fig. 4C–E). Upon infection with mutant GHs, the most increase in relative abundance was seen during infection with *ΔmalS*, *ΔmalS*, *ΔCHPNeu*, *ΔmalS*, *ΔglgX*, and *ΔmalS*, respectively for each of the glycans, which may suggest that these GHs targeted and degraded those glycans (Fig. 4C–E). Glycans m/z = 2167.777 (Hex_7_HexNAc_5_), 1722.645 (Hex_3_HexNAc_6_), 2953.047 (Hex_7_HexNAc_6_Neu5Ac_2_), 2776.020 (Hex_5_HexNAc_6_Fuc_3_Neu5Ac_1_), and 2556.946 (Hex_4_HexNAc_5_Fuc_4_Neu5Ac_1_) were accumulated (Fig. 4B–D), suggesting *Salmonella* induces the host to produce or increase the relative production of these glycans. Lastly, glycans m/z = 2688.004 (Hex_5_HexNAc_7_Fuc_3_) and 2483.904 (Hex_5_HexNAc_6_Fuc_1_Neu5Ac_1_) stayed at similar levels throughout the infection (Fig. 4B,D) suggesting that *Salmonella* does not have the specificity to recognize these glycans to degrade. These results suggest that the GHs within a genome contribute to virulence. Perhaps GHs represent a new virulence mechanism that is controlled by the host glycan structure and the gene content on the pathogen. This elucidates how glycan-degrading enzymes play an important role by altering the host glycan profiles during infection to gain access of the host epithelial cells. Also identifying exact glycans during infection will lead to identifying pathways that *Salmonella* uses to infect and invade the host.

## Discussion

Use of mucin and the glycocalyx by the microbiome is widely recognized^6^. Degrading the glycocalyx layer disrupts the host’s defensive barrier that provides access to the epithelial cell surface and receptors for binding, colonization, and invasion. Because *Salmonella* infections represent a persistent and major health challenge around the world it is important to understand how to control *Salmonella* infections. This study examined the specific enzymes used to degrade the glycan.

The sequence of events leading to *Salmonella* infection in the gut epithelial cells has been elucidated for the T3SS^22^. However, prior to T3SS (*invA*) access to the membrane, *Salmonella* must overcome the protective glycocalyx layer that coats the epithelium. In the gut *Salmonella* must first attach to and second degrade the host’s protective mucus and glycocalyx layer to gain access to the membrane and subsequently infect the underlying tissue. These results described here first identified that depletion of Neu5Ac from Caco-2 cells led to decreases in *Salmonella* association; therefore, this interaction lends insight into how *Salmonella* reaches the initial goals necessary to establish infection by using Neu5Ac as a potential receptor for adherence process.

In order to breach the glycocalyx layer, pathogens have evolved mechanisms to degrade the glycocalyx, which is common in many organisms^6^,^7^,^8^,^9^,^10^. *Salmonella* specifically is equipped with 48 enzymes from 21 families of glycosyl hydrolases to overcome this barrier. This study found expression of specific glycan-degrading enzymes in *Salmonella* used to degrade the glycocalyx and alter the host glycans to mediate invasion, which were previously unrecognized genes important to the virulence mechanism.

While the microbe is degrading the host glycan, it is also very important to study the host response during degradation. Efforts were made to improve our understanding of glycan degradation and alteration compexity in its entirety via the use of high resolution mass spectrometry^24^ and gene expression profiling. The host genes that hydrolyze mannose, fucose, and Neu5Ac from the glycan; as well as sialyltransferases, were induced during *Salmonella* infection (Fig. 3); supporting the hypothesis that new glycan structures were produced *de novo* (Fig. 2F) during infection. Hooper *et al*. and Bry *et al*. also found *in vivo* that the host produced new glycans containing fucosylation in the ileum that is microbe induced via α1,2-fucosyltransferase transcripts^6^,^7^. Refinement of the glycan by the *Salmonella* during infection was also observed (Fig. 4A). The shift to low Neu5Ac content is supported by these changes in sialidases and sialyltransferases (Fig. 3D,E, Supplementary Table S4). The accumulation of fucosylated glycans is supported by the changes in host fucosidases and fucosyltransferases (Fig. 3C) after infection, as previously observed^6^,^7^. Pickard *et al*. also observed that rapid fucosylation appears to be a protective mechanism that utilizes the host’s resources during host-microbe interactions during pathogen induced stress^25^. These observations confirm the host response to glycan modification during *Salmonella* LT2 association and suggest a dynamic shift in the glycan is caused by *Salmonella* degradation coupled with the host remodeling to produce a glycan of different structures as the infection progresses. This could be a protective mechanism for the host during infection and suggests that the host is actively producing new glycans in response to *Salmonella* infection. The mechanism dictating this phenomenon has yet to be elucidated, which requires additional experimentation to uncover the direct effect of *Salmonella* on host glycan metabolism. Glycan biosynthesis is significantly affected by the disease states and distinct glycan structures could provide information about the specific pathologic states of disease^23^. The host constantly remodels its glycans without altering its intrinsic function, which alters microbiome association^3^ and modulates host immune surveillance methods^26^. The host may alters its glycan composition on their cell surfaces to eliminate the expression of a terminal glycan structures in order to limit pathogen binding. The host may discard non-critical glycans to allow its survival^3^. The loss of a particular glycan may involve inactivation of one or more genes involved in glycan biosynthesis^27^ and can prevent recognition by pathogens using structure as a receptor. In an effort to alter its glycans to evade pathogens, the host may create new glycan structures either by synthesis or modification.

By combining microbial genomics, glycan profiling, and infection analyses, this study provides unprecedented molecular details for the role of *Salmonella* GHs’ during infection of Caco-2 cells. Alterations in the degree of branching, changes in the amount, linkage, and degrees of sialylation and fucosylation in *N*-glycans have been reported as a consequence of diseases^28^,^29^, but this is the first report to describe how *Salmonella* Typhimurium degraded the glycans that resulted in an infection. Deleting GHs used for glycan degradation resulted in invasion magnitude equal to those associated with T3SS in *Salmonella*, suggesting that these enzymes may be as important as secretion systems. Defining the virulence characteristics and diversity of GHs in *Salmonella* can provide new insights into the importance of glycan structures during host-pathogen interactions. This study further expands our understanding of the infection characteristics and may lead to the host response in glycan modification as mutualistic events between the host production and pathogen degradation. Some of the more encouraging prospects of this research are the potential for new treatments for gastroenteritis caused by *Salmonella*. This work may provide the basis of novel strategies to control enteric infections by targeting glycocalyx-degrading enzymes. The complexity of the pan-genome of *Salmonella*, as well as the multiple methods of infection used by *Salmonella*^30^,^31^ demands the use of multi-omics to define innovative targets to control infection. Use of antibiotics alone is not meeting the needs to control this organism and often leads to increased susceptibility to other pathogens^32^. Use of glycan degradation will preempt *Salmonella* from gaining access of the membrane – a novel method to control infection that may be of use in multiple pathogens in the gut and other epithelial surfaces.

## Methods

### Cell Culture

All cell *in vitro* experiments were done using colonic epithelial cells (Caco-2; ATCC HTB-37) as described previous^33^,^34^,^35^,^36^,^37^. Detailed description of the cell growth conditions is available in Supplementary Information.

### Bacterial Strains and Growth Conditions

*Salmonella enterica* subsp. *enterica* serovar Typhimurium strain LT2 (ATCC 700720; *Salmonella* WT) and the deletion mutants made in this study were used. All isolates were grown in LB (Difco, BD) at 37 °C with shaking at 220 rpm for 14–16 hours before use. Each biological replicate was done with a new vial of frozen stock after thawing and growth twice as described above.

### Gene Deletion

Bacterial gene deletions were done as described by Datsenko and Wanner^20^. Detailed description of gene deletions is available in Supplementary Information.

### Genomic sequencing and comparison

Each isolate was sequenced as described by Ludeke *et al*.^38^ as defined by the methods of the 100 K Pathogen Genome Sequencing Project^39^,^40^,^41^,^42^,^43^,^44^,^45^,^46^,^47^. Abyss 1.5.2 was used to assemble the paired end reads using k = 64^48^. Prokka was used for annotation^49^. Each genome was compared to the wild type by determining the genome distance using Genome-to-Genome Distance Calculator (GGDC) (http://ggdc.dsmz.de/distcalc2.php)^50^,^51^. Whole genome comparisons were done using Mauve under Progressive Mauve as described by Darling *et al*.^52^,^53^. Contigs were reordered using the *reorder contigs* option in Mauve with default parameters using *Salmonella* Typhimurium LT2 ATCC 700720 (accession number AE006468) as the reference genome. All raw genome sequences generated in this study are available in the NCBI SRA as part of the 100 K Pathogen Genome Project Bioproject Accession PRJNA186441. Accession numbers are listed in Table S5. Single gene analyses were done by extracting sequences from each genome and using MUSCLE through Geneious version 6.1.8 to align sequences^54^,^55^.

### Bacterial Association Measurements

Bacterial association was determined using a modified gentamycin protection assay^56^,^57^ after adding each *Salmonella* treatment in a 96-well plate containing an MOI of 1:1000. The assay was done after incubation for 60 min at 37 °C with 5% CO_2_. Adhered bacteria were measured after the cell culture medium was aspirated and the host/microbe cell mixture was washed once with 200 μl of 1X PBS buffer (pH 7). The host and associated microbes were lysed using 50 μl of commercial Warnex lysis buffer (AES Chemunex) for use in qPCR assays to determine the absolute amount of host and bacteria^57^. All assays were done in three biological replicates.

To calculate number of adhered bacteria, the mean of the number of invaded bacteria was subtracted from the mean of the total number of host associated bacteria. The error for adhered bacteria was propagated using equation (ΔZ)^2^ = (ΔA)^2^ + (ΔB)^2^ where ΔZ is the standard error of mean (SEM) for adhered bacteria, ΔA is SEM for total host associated bacteria and ΔB is SEM for invaded bacteria. One-way ANOVA with Tukey test was done to find significant differences across treatment’s and control’s group means.

### Gene Expression during Infection

Samples were collected as previously described^32^,^34^,^58^,^59^. Briefly, Caco-2 cells were cultured in T-75 flasks (BD) and were serum starved 24 h before infection. Respective bacterial treatments with *Salmonella* WT and the deletion mutants, at MOI of 1000, were used to infect epithelial cells as described previously. All treatments were incubated at 37 °C with 5% CO_2_ for 60 min. 10 ml of TRIzol LS reagent (Invitrogen, Carlsbad, CA) was added to the cells and mixed with pipette followed by centrifugation at 7,200 × g for 5 min to pellet the host associated bacteria. TRIzol LS supernatant was stored in a clean tube and further processed for RNA extraction from infected Caco-2 cells. The bacterial pellet was suspended in 2 ml of fresh TRIzol LS, gently mixed and further processed for RNA extraction from host associated bacteria. The experiment was done in two biological replicates.

#### Bacterial RNA extraction and gene expression

Sample preparation for gene expression profiling was performed with RNA isolation, which was done using TRIzol LS reagent (Invitrogen) as described previously^32^,^34^. Total RNA (10 μg in 20 μl) was reverse transcribed into cDNA with 6 μg of random hexamers and 400 U of Superscriptase II (Invitrogen) according to the manufacturer’s protocol. The reaction mixture was cleaned by using the Qiaquick-PCR purification kit (Qiagen, Valencia, CA) according to the manufacturer’s instructions and as described previously^34^. The purified single-strand cDNA was eluted from the columns twice with a total of 100 μl of nuclease free water (Ambion, Austin, TX). cDNA fragmentation was done using 0.6 U of DNaseI (Promega, Madison, WI) per μg of cDNA, according to the instructions. The fragmented 1 μg of cDNA was labeled using 2 μl of GeneChip DNA Labeling reagent (Affymetrix, Santa Clara, CA) and 60 U of Terminal Transferase enzyme (New England Biolabs, Ipswich, MA). The samples were denatured prior to hybridization, at 98 °C for 10 min followed by snap cooling at 4 °C for 5 min.

#### Hybridization and normalization

Labeled cDNA was hybridized onto a custom made Affymetrix GeneChip designed against all the annotated coding sequences of *Salmonella* LT2 ATCC 700720 (*Salmonella* WT)^32^,^34^,^60^. The chips were hybridized and scanned at the Center for Integrated BioSystems (Utah State University, Logan, UT) as per manufacturer’s protocols. Raw data (.cel files) was background corrected, quantile normalized and summarized using MS-RMA^32^,^61^. The resultant normalized Log_2_ transformed intensity matrix was used for further statistical analysis.

#### Caco-2 RNA extraction and gene expression

The TRIzol LS samples containing infected or non infected Caco-2 cells were frozen (Liquid N_2_) and thawed (70 °C) twice. To 750 μl of TRIzol LS sample, 250 μl of water was added. This was further processed for RNA extraction using manufacturer’s (TRIzol LS, Invitrogen) instructions. Synthesis of cDNA, biotin labeled cRNA, fragmentation and purification of cRNA were carried out using one-cycle cDNA synthesis kit (Affymetrix, Santa Clara, CA).

#### Host hybridization and normalization

Labeled and fragmented cRNA (10 μg) was hybridized onto the Affymetrix HGU133Plus2 GeneChips as per manufacturer’s recommendations at the Center for Integrated BioSystems (Utah State University, Logan, UT). Raw data (.cel files) was background corrected; quantile normalized and summarized using RMA. RMA normalized data was then filtered through the PANP algorithm to make presence-absence calls for each probe set. Probe sets that were called present in at least one of the samples were included in further statistical analysis.

#### Statistical analysis for gene expression

Gene expression profiles for *Salmonella* WT alone and in the presence of the epithelial cells were obtained 60 min post infection. The data was analyzed as two class unpaired with T statistic, using Significance Analysis of Microarrays (SAM)^62^. All the genes were ranked based on the score from SAM output. This pre-ordered ranked gene list was then used in Gene Set Enrichment Analysis software (GSEA) to detect the coordinate changes in the expression of groups of functionally related genes, upon respective treatments. The gene sets were defined based on the annotations from Comprehensive Microbial Resource (CMR), Cluster of Orthologous Groups of proteins (COGs), and Virulence Factors of pathogenic bacteria Data Base (VFDB).

### Glycan Degradation

Differentiated Caco-2 cells in T-75 flasks were infected with *Salmonella* WT and its knockouts at MOI 1:1000 and incubated for 60 min at 37 °C, 5% CO_2_. Infection samples were washed three times with ice cold 1X PBS to remove non-adherent bacteria and cellular debris. Cells were scraped from the flask with cell scraper and were kept on ice until cell membrane extraction and *N*-glycan release as detailed in Supplemental Information online. *N*-glycans were enriched and analyzed using Agilent HPLC-Chip-QTOF MS (Agilent, CA) as detailed in the Supplementary Information. *N*-Glycans were identified by composition with a retrosynthetic library using accurate mass according to mass tolerance, retention times, and abundance information^24^ and further verified by tandem MS (Supplementary Figure S6).

### Ingenuity Pathway Analysis

QIAGEN’S Ingenuity Pathway Analysis (IPA, QIAGEN Redwood City, www.qiagen.com/ingenuity) software was used to determine biological pathways associated with our gene expression data (Ingenuity Systems, http://www.ingenuity.com, IPA release version summer 2014) similar to He *et al*.^33^. Networks representing molecular interaction were constructed based on the IPA database. Determination of pathway associations was determined through IPA (Fisher’s exact test).

## Additional Information

**How to cite this article**: Arabyan, N. *et al*. *Salmonella* Degrades the Host Glycocalyx Leading to Altered Infection and Glycan Remodeling. *Sci. Rep.* **6**, 29525; doi: 10.1038/srep29525 (2016).

## Supplementary Material

Supplementary Information
