# Supplementary material for: Salmonella Degrades the Host Glycocalyx Leading to Altered Infection and Glycan Remodeling
Source: Sci Rep. 2016 Jul 8;6:29525. doi: 10.1038/srep29525 (PMC4937416; doi:10.1038/srep29525)
Supplement: Supplementary Information [file srep29525-s1.pdf]

Supplementary Information

Salmonella Degrades the Host Glycocalyx Leading to Altered Infection and Glycan Remodeling

Narine Arabyan[1], Dayoung Park[2], Soraya Foutouhi[1], Allison M. Weis[1], Bihua C. Huang[1], Cynthia C. Williams[2], Prerak Desai[1†], Jigna Shah[1‡], Richard Jeannotte[1£,4], Nguyet Kong[1], Carlito B. Lebrilla[2,3], and Bart C. Weimer[1*]

Supplementary Figures

A

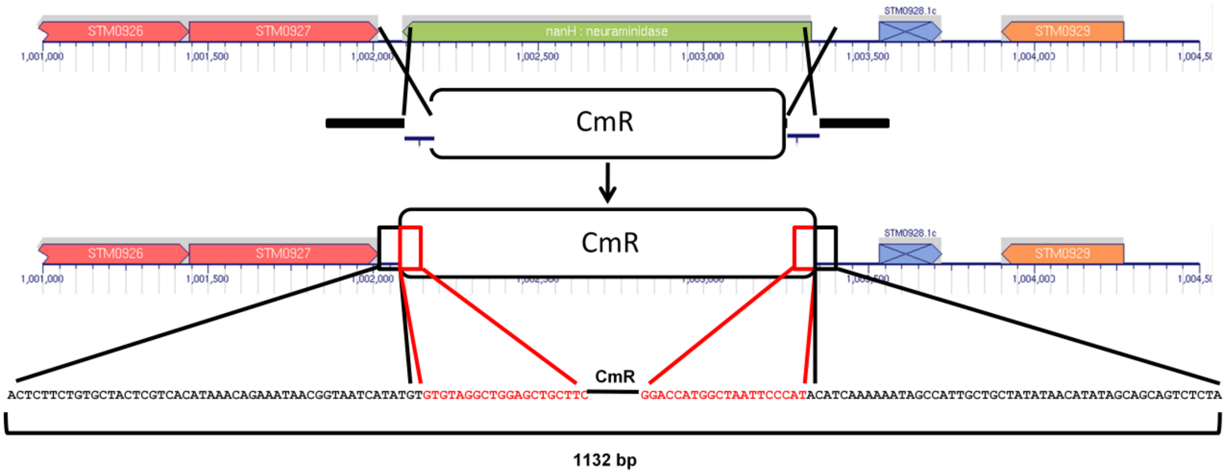

	WT Gene	WT Junction	KO Gene	KO Junction
melA	+	-	-	+
nanH	+	-	-	+
CHPNeu	+	-	-	+
malS	+	-	-	+
glgX	+	-	-	+

B

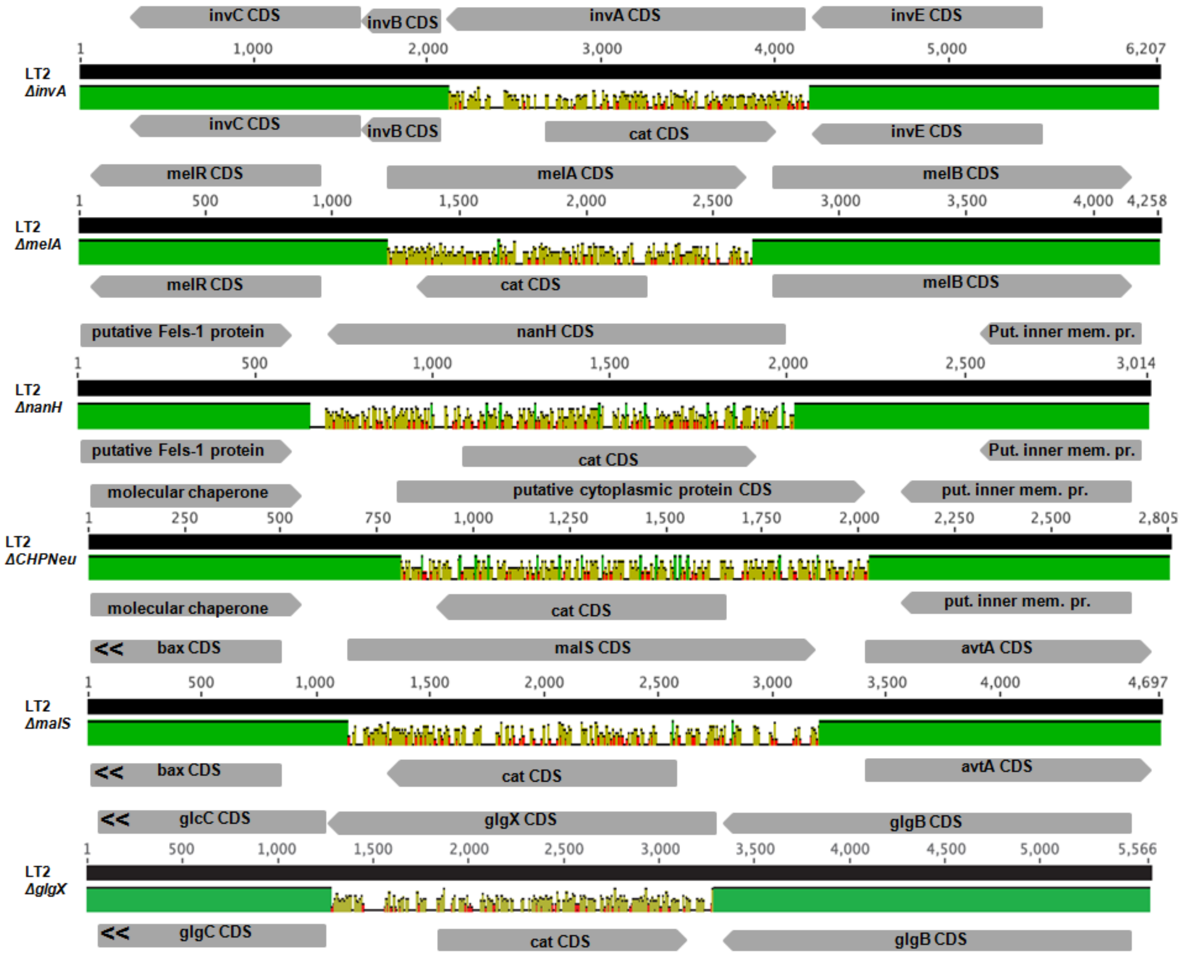
invC CDS
invB CDS
invA CDS
invE CDS
LT2
ΔinvA
cat CDS
melR CDS
melA CDS
melB CDS
LT2
ΔmelA
cat CDS
putative Fels-1 protein
nanH CDS
Put. inner mem. pr.
LT2
ΔnanH
cat CDS
molecular chaperone
putative cytoplasmic protein CDS
put. inner mem. pr.
LT2
ΔCHPNeu
cat CDS
bax CDS
malS CDS
avtA CDS
LT2
ΔmalS
cat CDS
glcC CDS
glgX CDS
glgB CDS
LT2
ΔglgX
glgC CDS
cat CDS

C

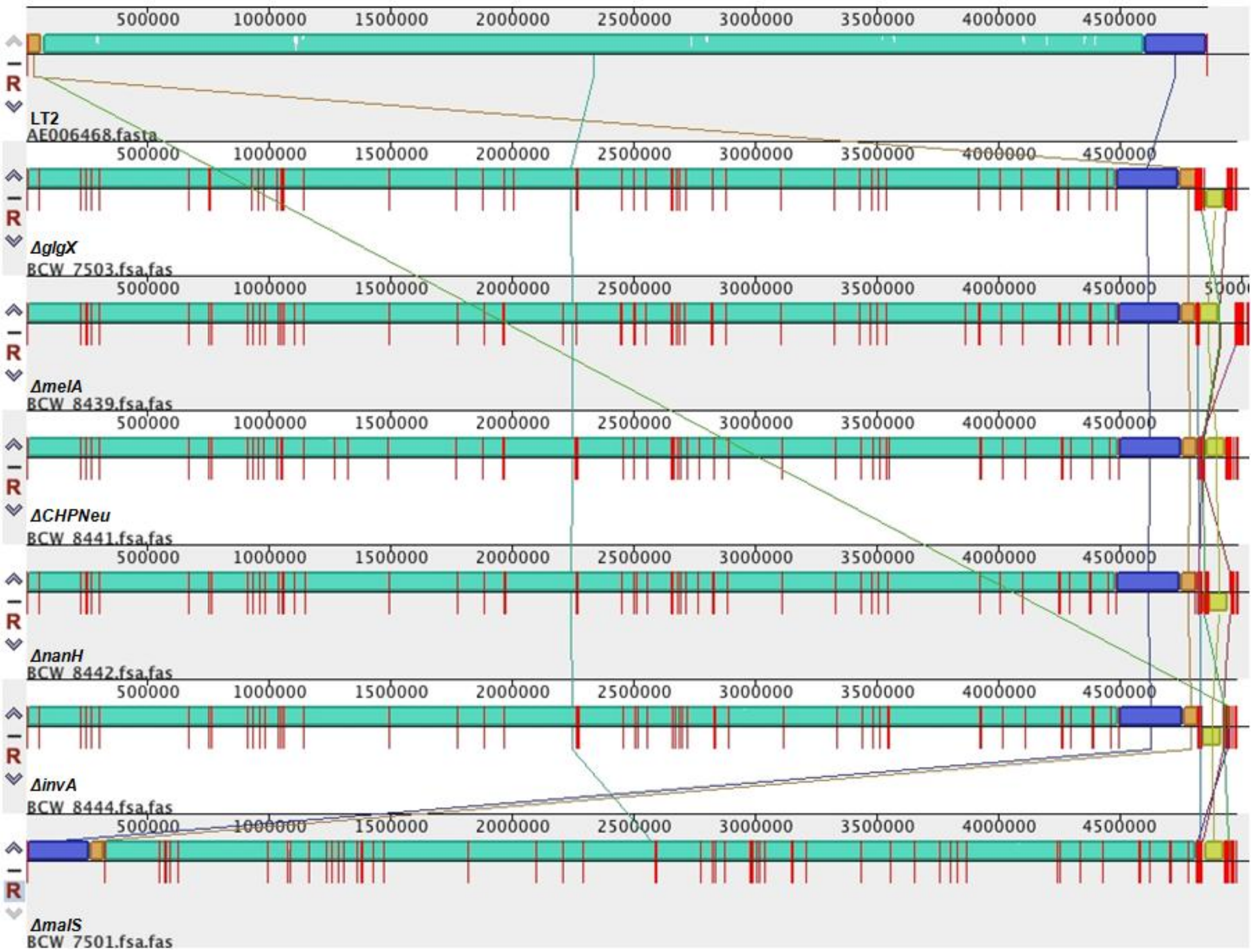

Supplementary Figure S1. Generation of specific deletions in *Salmonella* LT2. (**A**) The schematic of targeted gene deletion. Here, *nanH* gene is depected as an example. Two 70-mer primers amplify the region containing the chloramphenicol resistance cassette (CmR) from plasmid pKD3. The resulting linear PCR product has 50 bases at each end that are homologous to the sequence of *nanH*. Transformation of this PCR product into *Salmonella* LT2 that expresses λ-Red recombinase leads to homologous recombination event resulting in the exchange of the *nanH* with CmR. All mutant strains used in this study were generated in the same way. PCR results of each deletion are summarized in the schematic. (**B**) Single gene analyses using MUSCLE to determine deletion of target gene and insertion of *cat* (Chloramphenicol acetyltransferase). The gene of interest and the neighboring area were extracted from each genome compared to the wild type to verify the lack of rearrangement and the targeted double cross over replacement. In each comparison, the black track

represents the segment of interest in the wild type and green track represents the corresponding segment in the deletion strain. The absence of the gene of interest and the presence of the chloramphenicol resistance gene (*cat* CDS) in the knockout strain verified the targeted distruption. The gene (CDS) arrangement for each comparison is shown (gray bars). The upstream and downstream regions have 100% homology within the neighborhood. (**C**) Mauve visualisation of whole genome alignment and comparison. *Salmonella* Typhimurium LT2 was used as the reference genome (top track) and compared to each of the individual deletion genomes. This analysis showed genome block homology and gene neighborhood conservation. Reference based assembly also showed that only the gene of interest was replaced with the chloramphenicol resistance gene. Both analyses (de nove and reference based assemblies) found that these genomes were syntenic.

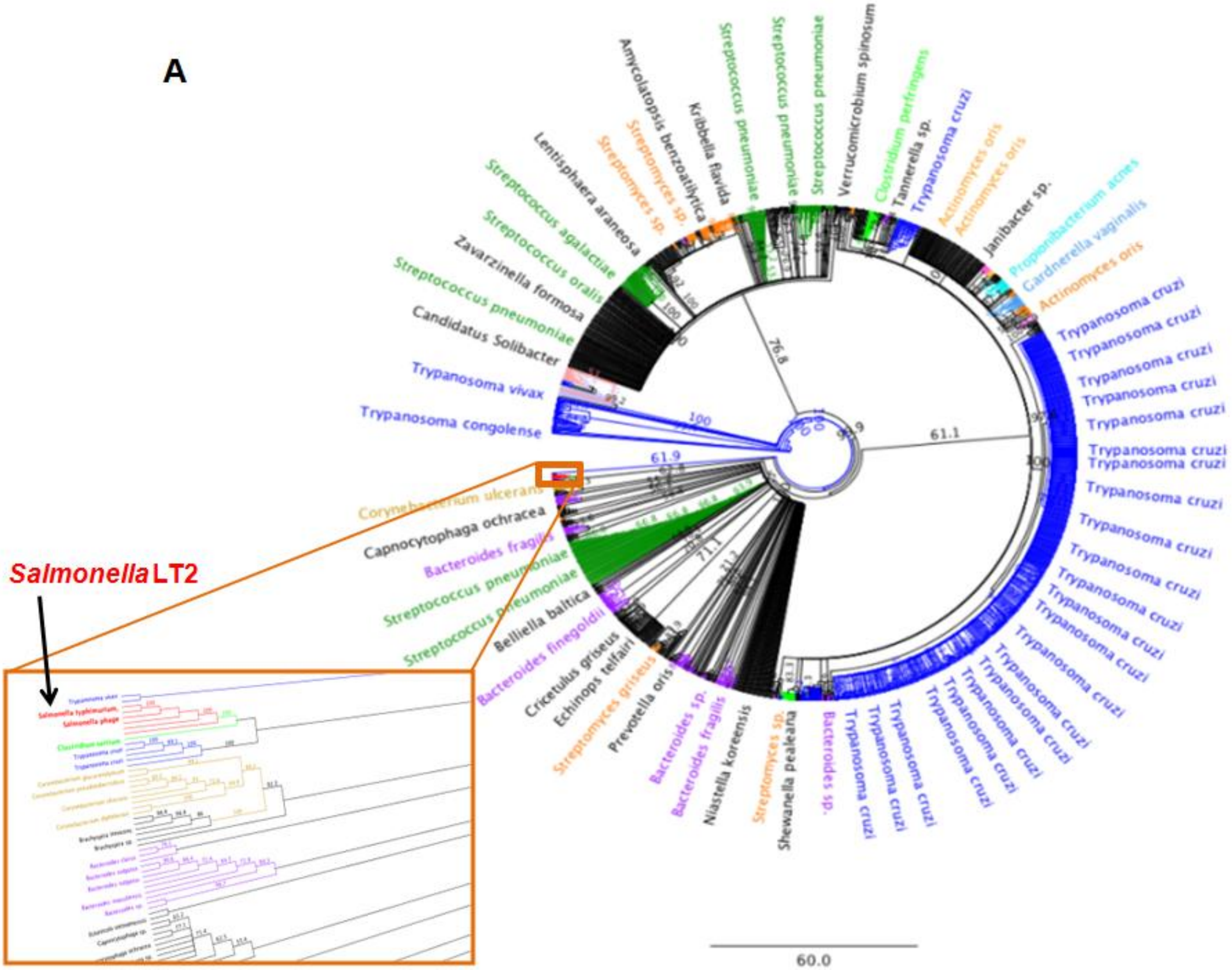
A
Salmonella LT2
Amycolatopsis benzoatilytica
Kribbella flavida
Streptococcus pneumoniae
Verrucomicrobium spinosum
Clostridium perfringens
Tannerella sp.
Trypanosoma cruzi
Actinomyces oris
Janibacter sp.
Propionibacterium acnes
Gardnerella vaginalis
Streptomyces sp.
Lentisphaera araneosa
Streptococcus agalactiae
Streptococcus oralis
Zavarzinella formosa
Candidatus Solibacter
Trypanosoma vivax
Trypanosoma congolense
Corynebacterium ulcerans
Capnocytophaga ochracea
Bacteroides fragilis
Belliella baltica
Bacteroides finegoldii
Cricetulus griseus
Echinops telfairi
Streptomyces griseus
Prevotella oris
Bacteroides sp.
Niastella koreensis
Shewanella pealeana
76.8
61.1
61.9
60.0

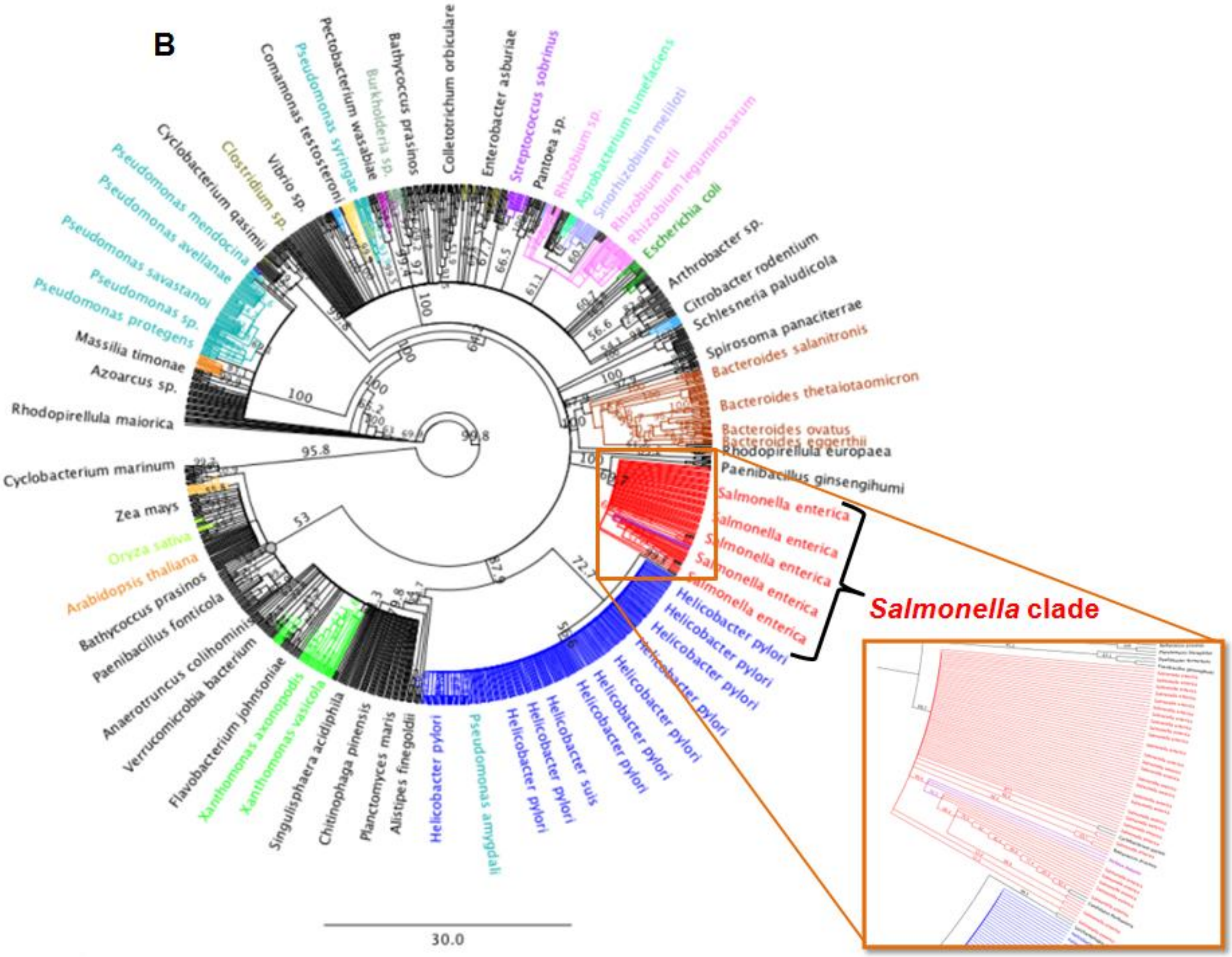

Supplementary Figure S2. Diversity of sialidases in nature. Phylogenetic trees of *nanH* (A) and *CHPNeu* (B). This study bioinformatically examined the sialidase diversity and genomic distribution among microbes. Phylogenetic trees were constructed using a Neighbor-Joining consensus tree with 1000 bootstrap replicates. This analysis revealed that members of the same genera (indicated with the same color) were found on different sialidase clades (*Escherichia coli*, *Klebsiella* spp., *Bacillus* spp., *Pseudomonas* spp., *Helicobacter* spp., *Bacteroides* spp., *Clostridium* spp., *Streptococcus* spp., *Salmonella* spp., *Rumonococcus* spp.), indicating widespread horizontal gene transfer (HGT). *CHPNeu* and *nanH* were widely distributed among pathogenic and commensal bacteria and parasites. *nanH* was most closely related to *Clostridium perfringens*, suggesting that *Salmonella* Typhimurium LT2 acquired this gene as a result of HGT. The direct impact of multiple sialidases remains to be explored. Each color represents different genera. Bootstrap values are listed on each branch.

Nodes represent speciation events. Branch length represents the degree of evolutional changes over time. The scale bar at the bottom of each tree is nucleotide substitutions per site.

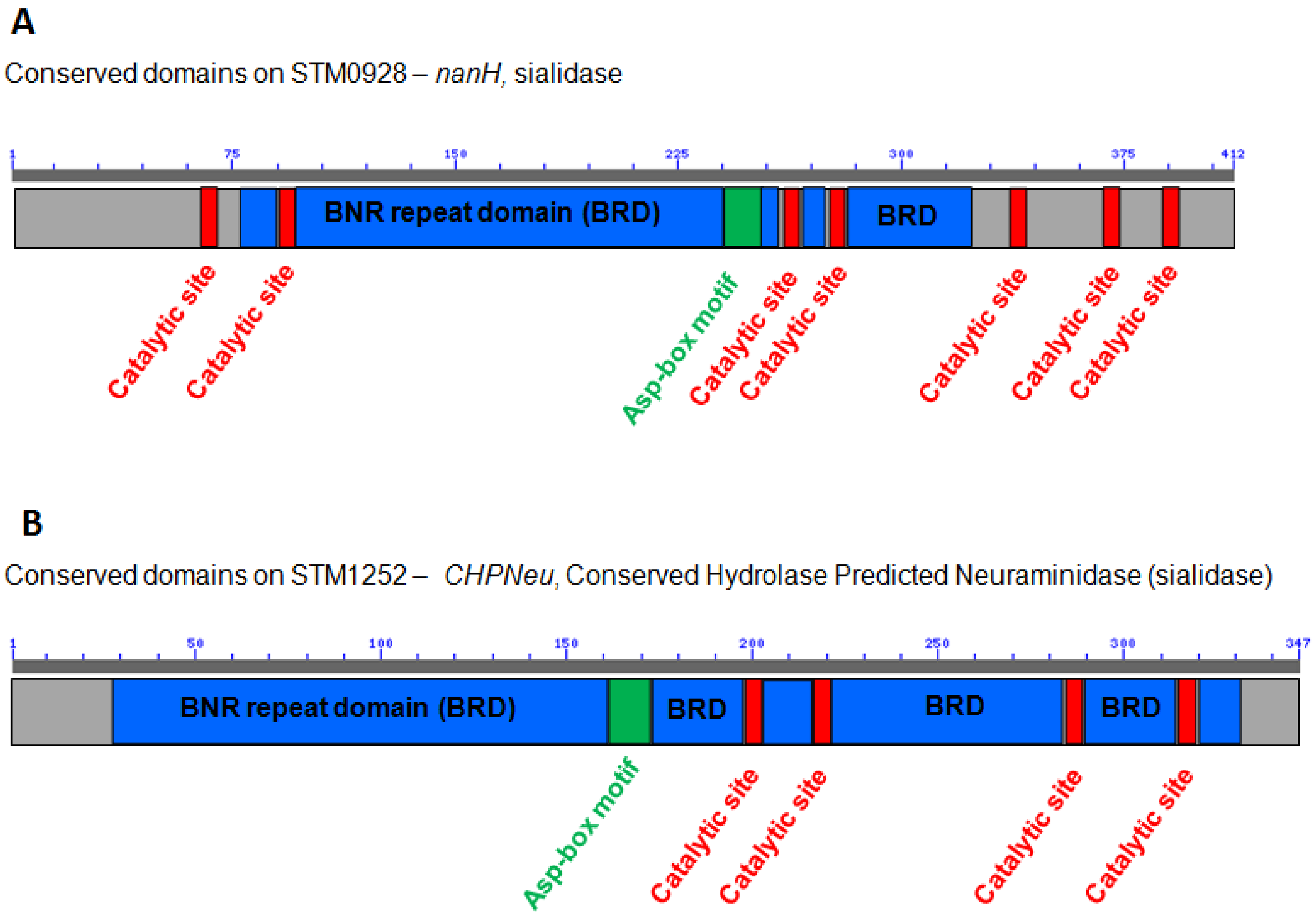

Supplementary Figure S3. Comparison of sialidases *nanH* and *CHPNeu*. The two sialidases from *Salmonella enterica* Typhimurium LT2 have the same domains and function as sialidases. The conserved regions in *nanH* (A) and *CHPNeu* (B) include an Asp-box motifs (with conserved residues: Ser/Thr-X-Asp-[X]-Gly-X-Thr- Trp/Phe) and a BNR repeat-like domain that suggests it may act as a sialidase, however they are structurally very different. While the function and domains of sialidases demonstrate homology; structural and domain organization demonstrate that these genes have undergone domain shuffling or may be orthologs. Domain shuffling is not a common event in proteins that are important for infection and are under strong diversifying selection pressures. This difference led to different invasion phenotypes during the *in vitro* infection of Caco-2 cells.

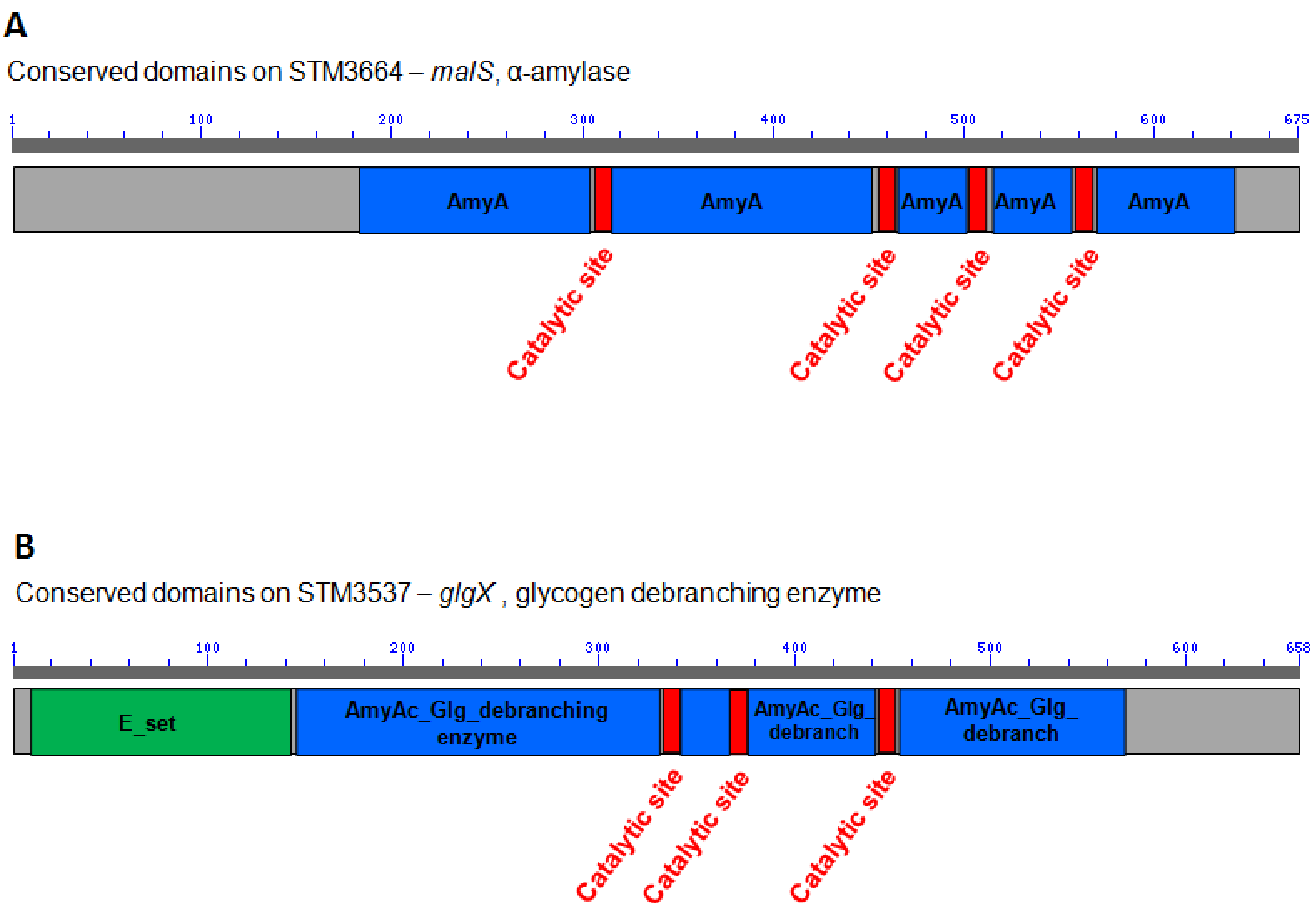

Supplementary Figure S4. Comparison of amylases *malS* and *glgX*. The two amylase-like enzymes in *Salmonella* LT2 also showed opposite results. Both enzymes had similar domains, however, both had structural variations. (A) *malS* is composed of an α-amylase domain and four catalytic sites. (B) The glycogen debranching enzyme, glgX, contains an α-amylase catalytic domain. On N-terminal side, glgX has an E or early set domain that is associated with the catalytic domain of the Glycogen debranching enzyme and bacterial isoamylase.

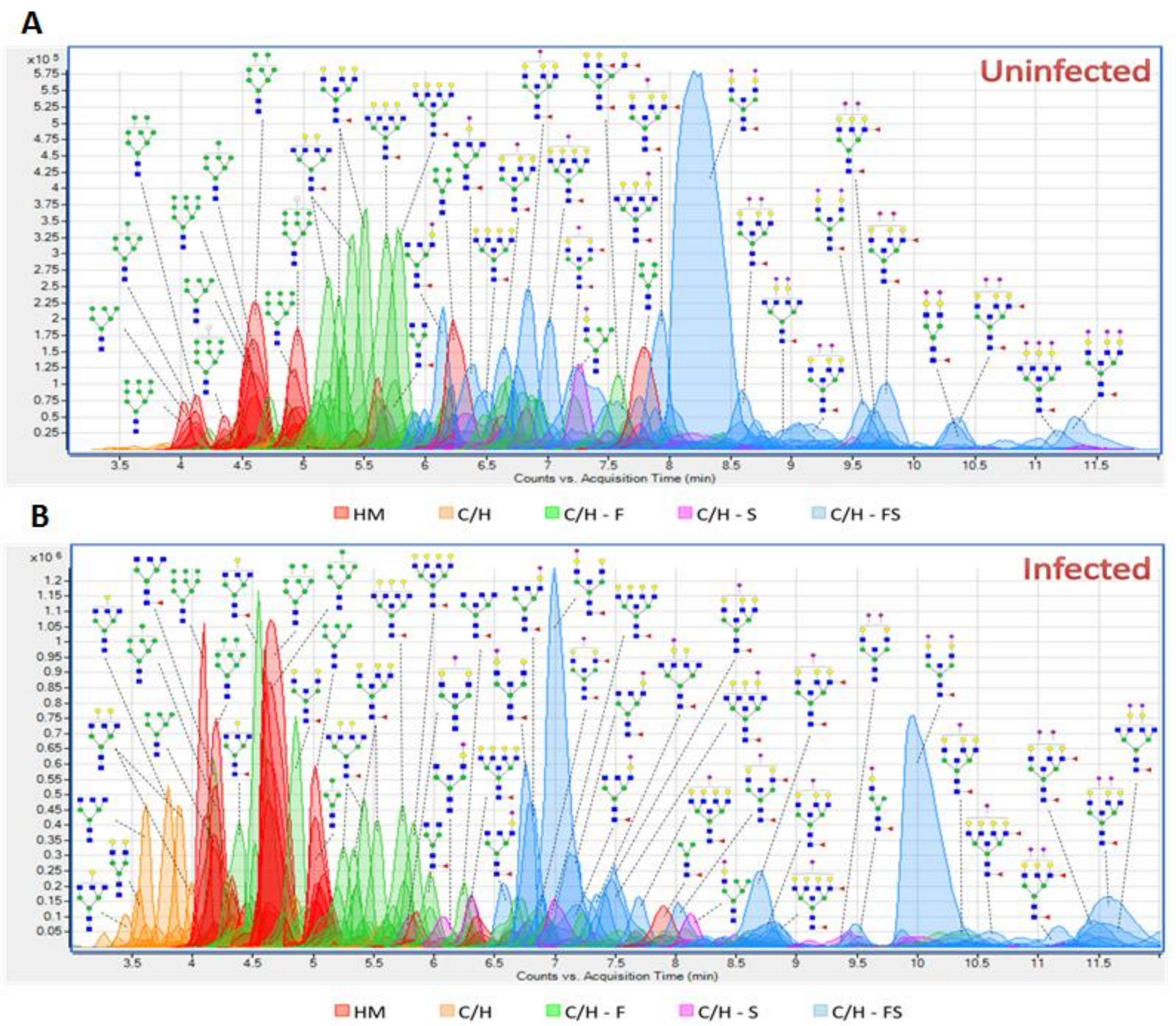

Supplementary Figure S5. Glycan profiles. (A) An extracted compound chromatogram of counts vs. retention time is shown for uninfected Caco-2 cells. Each peak corresponds to a glycan which are colored distinguish between high mannose, neutral (no decorations) complex/hybrid, fucosylated, sialylated, and both fucosylated & sialylated glycans. (B) Glycan compositions found in Caco-2 after 1 hour of infection with *Salmonella* LT2. Select glycan structures that change during the course of infection have been assigned with putative structures.

A

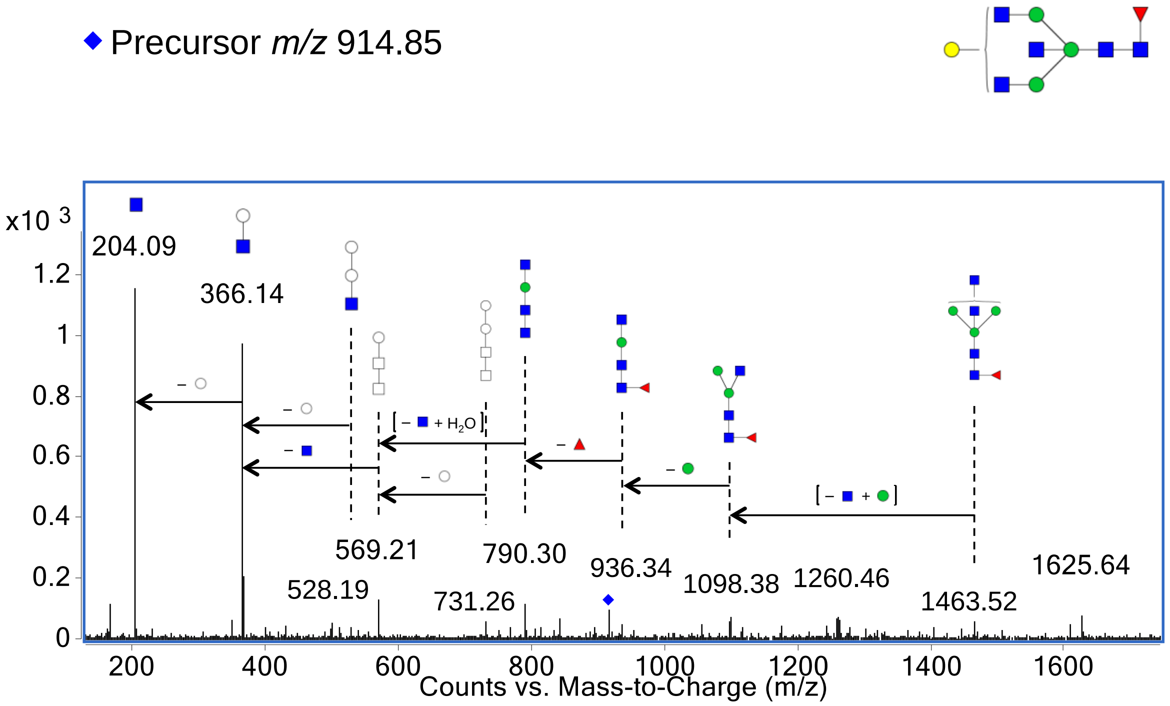

B

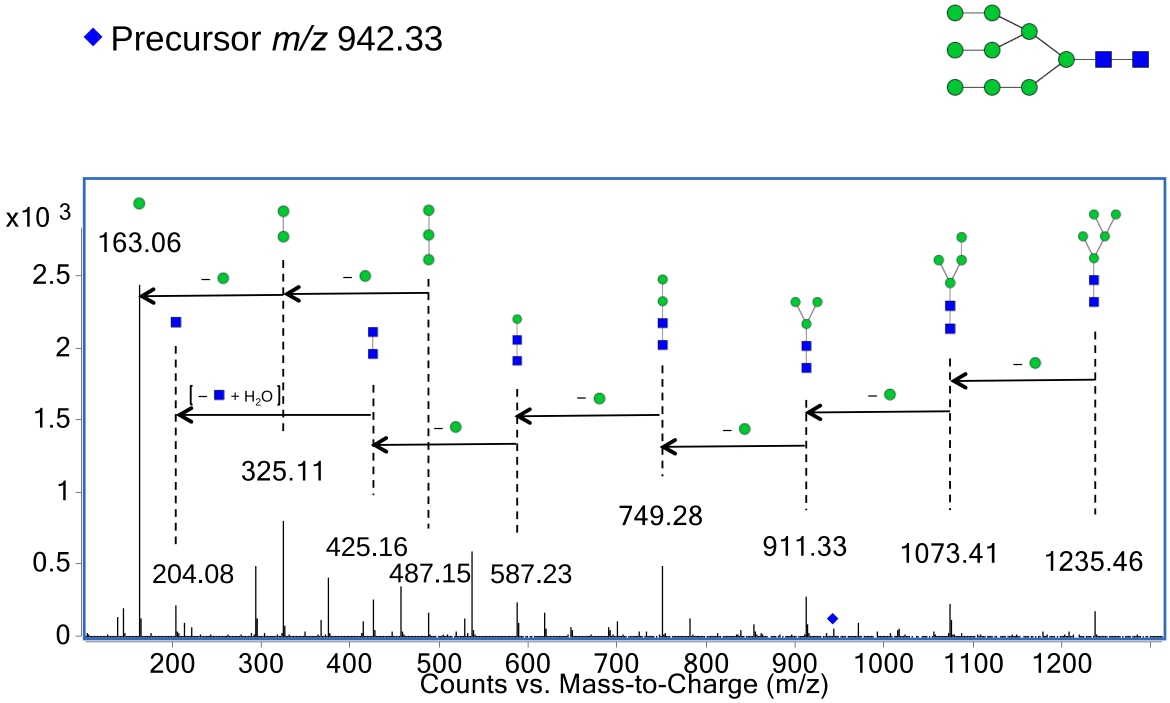

Supplementary Figure S6. Representative MS/MS spectra of identified *N*-glycan structures. (A-B) Sequential fragment losses are annotated inset. Precursor ions are indicated by a blue diamond.

Supplementary Tables

Supplementary Table S1: Gene expression intensity of *Salmonella* LT2 glycosyl hydrolases within 60 minutes of infection of Caco-2 cells.

**Gene ID**	**Gene**	**Gene Name**	**Intensity of *Salmonella* WT**	**Intensity of Caco-2 + *Salmonella* WT**
STM4298	*melA*	alpha-galactosidase	4.490	2.978
STM1252	STM1252	putative cytoplasmic protein	6.120	5.658
STM0928	*nanH*	Neuraminidase	5.670	4.769
STM3664	*malS*	periplasmic alpha-amylase precursor	2.492	2.632
STM3537	*glgX*	glycogen debranching enzyme	3.629	4.328
STM2166	*bglX*	periplasmic beta-D-glucoside glucohydrolase	3.991	3.839
STM1209	*nagZ*	beta-hexosaminidase	4.401	4.711
STM1963	*amyA*	cytoplasmic alpha-amylase	3.255	3.581
STM3663	*bax*	putative ATP-binding protein	5.320	5.934
STM0260	*dniR*	transcriptional regulator	3.356	4.415
STM1799	*emtA*	membrane-bound lytic murein transglycosylase E	3.638	4.719
STM3617	STM3617	endo-1,4-D-glucanase	3.258	3.674
STM1242	*envE*	putative envelope protein	5.760	5.661
STM1182	*flgJ*	flagellar biosynthesis protein	3.712	4.266
STM3538	*glgB*	glycogen branching enzyme	4.049	5.440
STM3513	*malQ*	4-alpha-glucanotransferase	3.982	4.521
STM0401	*malZ*	maltodextrin glucosidase	4.225	3.804
STM2988	*mltA*	membrane-bound lytic murein transglycosylase A	4.284	4.345
STM3112	*mltC*	membrane-bound lytic murein transglycosylase C	3.136	4.196
STM0016	STM0016	hypothetical protein	2.984	3.492
STM0018	STM0018	putative exochitinase	4.148	3.981
STM0148	STM0148	putative cytoplasmic protein	4.201	3.573
STM0233	STM0233	putative endochitinase	3.976	3.319
STM0907	STM0907	putative chitinase	4.920	4.342
STM1316	*celF*	phospho-beta-glucosidase/cellobiose-6-phosphate hydrolase	3.848	4.216
STM1558	STM1558	putative glycosyl hydrolase	4.235	4.913
STM1559	STM1559	putative glycosyl hydrolase	3.700	4.042
STM1560	STM1560	putative alpha amylase	4.564	3.429
STM1869A	STM1869A	hypothetical protein	3.520	3.360
STM1911	STM1911	putative cytoplasmic protein	4.076	4.975
STM2612	STM2612	morphogenesis-like protein	3.866	3.938
STM2715	STM2715.S	probable prophage lysozyme	5.393	4.265

STM2831	*mltB*	membrane-bound lytic murein transglycosylase B	3.973	4.662
STM3051	*bglA*	6-phospho-beta-glucosidase A	4.780	5.603
STM3605	STM3605	putative phage endolysin	3.335	2.892
STM3679	STM3679	putative cytoplasmic protein	3.525	3.644
STM3775	STM3775	putative glycosyl hydrolase	3.990	3.874
STM4217	STM4217	putative soluble lytic murein transglycosylase	4.676	4.061
STM4426	*srfJ*	lysosomal glucosyl ceramidase-like protein	4.347	4.518
STM4582	*slt*	soluble lytic murein transglycosylase	3.961	3.411
STM1796	*treA*	Trehalase	3.190	3.920
STM4453	*treC*	trehalose-6-phosphate hydrolase	4.214	4.101
STM3603	*treF*	cytoplasmic trehalase	5.309	5.165
STM2567	*yfhD*	putative periplasmic amino acid-binding protein	3.314	4.280
STM3749	*yicI*	putative alpha xylosidase	4.818	4.449
STM4019	*yihQ*	putative alpha-xylosidase	3.913	3.818

Supplementary Table S2: Glycan found on Caco-2 cells and their changes during *Salmonella* infection. Ratios greater than 1 means increase in glycan abundance during infection, less than 1 means deacrease in glycan abundance during infection, ratios of about 1 means that glycan abundances did not change during infection, ratios equal to 0 means glycans disappeared during infection, and lastly, ratios that were undefined means that these are new glycans biosynthesized *de novo*. C = complex, H = hybrid, F = fucosylated, S = sialylated, and FS = fucosylated and sialylated.

	Theoretical Glycan Mass*	Glycan Composition	Class	Ratio of Infected / Uninfected	Structure
1	2061.751	$Hex_4HexNAc_4Fuc_2Neu5Ac_1$	C-FS	28.513	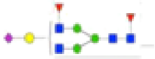
2	2953.047	$Hex_7HexNAc_6Neu5Ac_2$	C-S	22.386	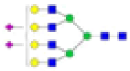
3	2776.020	$Hex_5HexNAc_6Fuc_3Neu5Ac_1$	C-FS	5.334	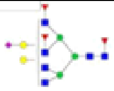
4	2363.898	$Hex_3HexNAc_7Fuc_3$	C-F	5.280	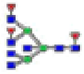
5	1519.566	$Hex_3HexNAc_5$	C	4.899	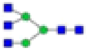
6	2556.946	$Hex_4HexNAc_5Fuc_4Neu5Ac_1$	C-FS	4.143	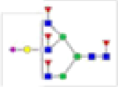
7	2280.825	$Hex_5HexNAc_5Fuc_1Neu5Ac_1$	C/H-FS	3.199	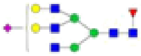
8	2692.946	$Hex_7HexNAc_4Fuc_1Neu5Ac_2$	H-FS	3.106	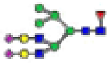
9	1681.619	$Hex_4HexNAc_5$	C/H	2.962	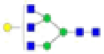
10	2938.073	$Hex_6HexNAc_6Fuc_3Neu5Ac_1$	C-FS	2.811	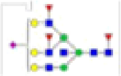
11	2734.993	$Hex_6HexNAc_5Fuc_3Neu5Ac_1$	C/H-FS	2.808	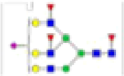
12	1925.725	$Hex_3HexNAc_7$	C	2.769	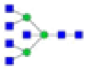
13	2014.761	$Hex_3HexNAc_6Fuc_2$	C-F	2.505	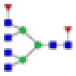
14	2167.777	$Hex_7HexNAc_5$	H	2.341	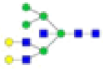
15	3099.105	$Hex_7HexNAc_6Fuc_1Neu5Ac_2$	C-FS	2.219	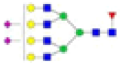
16	1316.487	$Hex_3HexNAc_4$	C	2.168	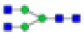
17	1843.672	$Hex_5HexNAc_5$	C/H	2.126	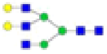
18	1722.645	$Hex_3HexNAc_6$	C	2.022	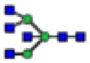
19	2702.979	$Hex_6HexNAc_7Neu5Ac_1$	C-S	2.022	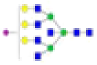

20	1884.698	$Hex_4HexNAc_6$	C	2.018	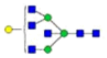
21	2160.819	$Hex_3HexNAc_6Fuc_3$	C-F	1.985	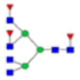
22	2071.783	$Hex_3HexNAc_7Fuc_1$	C-F	1.940	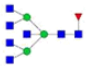
23	1558.539	$GlcNAc_2Man_7$	HM	1.803	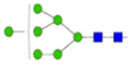
24	2996.115	$Hex_6HexNAc_7Fuc_4$	C-F	1.792	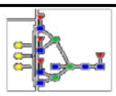
25	1624.597	$Hex_4HexNAc_4Fuc_1$	C/H-F	1.786	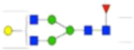
26	1478.539	$Hex_4HexNAc_4$	C/H	1.738	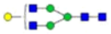
27	2379.893	$Hex_4HexNAc_7Fuc_2$	C-F	1.733	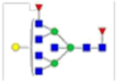
28	2937.052	$Hex_6HexNAc_6Fuc_1Neu5Ac_2$	C-FS	1.707	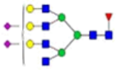
29	1072.381	$GlcNAc_2Man_4$	HM	1.694	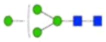
30	2686.984	$Hex_5HexNAc_7Fuc_1Neu5Ac_1$	C-FS	1.641	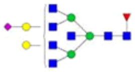
31	1712.613	$Hex_4HexNAc_3Fuc_1Neu5Ac_1$	C-FS	1.614	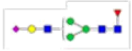

225

32	2733.973	$Hex_6HexNAc_5Fuc_1Neu5Ac_2$	C/H-FS	1.515	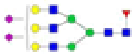
33	910.328	$GlcNAc_2Man_3$	HM	1.501	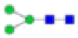
34	1234.433	$GlcNAc_2Man_5$	HM	1.495	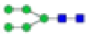
35	2718.999	$Hex_5HexNAc_5Fuc_4Neu5Ac_1$	C-FS	1.473	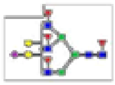
36	1720.592	$GlcNAc_2Man_8$	HM	1.470	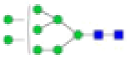
37	1665.624	$Hex_3HexNAc_5Fuc_1$	C-F	1.384	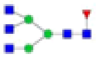
38	1396.486	$GlcNAc_2Man_6$	HM	1.356	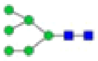
39	1113.407	$Hex_3HexNAc_3$	C	1.356	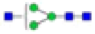
40	1882.645	$GlcNAc_2Man_9$	HM	1.339	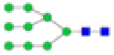
41	1973.735	$Hex_4HexNAc_5Fuc_2$	C/H-F	1.334	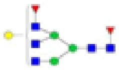
42	1827.677	$Hex_4HexNAc_5Fuc_1$	C/H-F	1.326	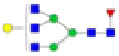
43	1802.645	$Hex_6HexNAc_4$	H	1.308	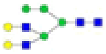
44	3302.185	$Hex_7HexNAc_7Fuc_1Neu5Ac_2$	C-FS	1.298	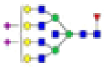
45	2118.772	$Hex_4HexNAc_5Fuc_1Neu5Ac_1$	C-FS	1.293	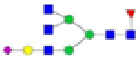
46	1989.729	$Hex_5HexNAc_5Fuc_1$	C/H-F	1.281	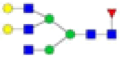
47	1437.513	$Hex_5HexNAc_3$	H	1.254	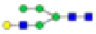
48	2369.861	$Hex_5HexNAc_4Fuc_3Neu5Ac_1$	C/H-FS	1.149	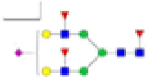
49	2321.851	$Hex_4HexNAc_6Fuc_1Neu5Ac_1$	C-FS	1.137	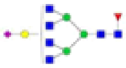
50	2792.015	$Hex_6HexNAc_6Fuc_2Neu5Ac_1$	C-FS	1.117	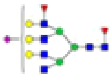
51	2483.904	$Hex_5HexNAc_6Fuc_1Neu5Ac_1$	C-FS	1.117	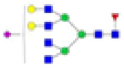

226

52	2087.778	$Hex_4HexNAc_7$	C	1.108	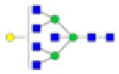
53	2217.841	$Hex_3HexNAc_7Fuc_2$	C-F	1.097	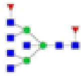
54	2134.767	$Hex_5HexNAc_5Neu5Ac_1$	C/H-S	1.094	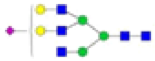
55	2865.031	$Hex_7HexNAc_7Neu5Ac_1$	C-S	1.091	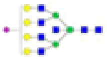
56	2046.751	$Hex_5HexNAc_6$	C	1.059	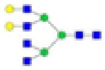
57	1275.460	$Hex_4HexNAc_3$	C/H	1.057	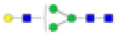
58	2223.803	$Hex_5HexNAc_4Fuc_2Neu5Ac_1$	C/H-FS	1.052	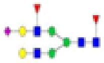
59	1640.592	$Hex_5HexNAc_4$	C/H	1.045	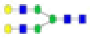
60	2688.004	$Hex_5HexNAc_7Fuc_3$	C-F	1.042	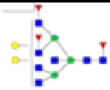
61	1915.693	$Hex_4HexNAc_4Fuc_1Neu5Ac_1$	C-FS	1.033	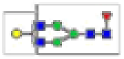
62	3157.147	$Hex_7HexNAc_7Fuc_2Neu5Ac_1$	C-FS	1.010	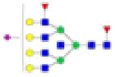
63	3303.205	$Hex_7HexNAc_7Fuc_3Neu5Ac_1$	C-FS	1.008	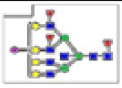
64	2995.094	$Hex_6HexNAc_7Fuc_2Neu5Ac_1$	C-FS	1.000	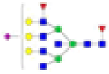
65	1462.544	$Hex_3HexNAc_4Fuc_1$	C-F	0.998	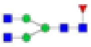
66	1745.623	$Hex_6HexNAc_3Fuc_1$	H-F	0.995	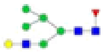
67	2629.962	$Hex_5HexNAc_6Fuc_2Neu5Ac_1$	C-FS	0.990	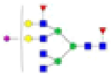
68	1868.703	$Hex_3HexNAc_6Fuc_1$	C-F	0.972	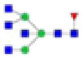
69	1972.714	$Hex_4HexNAc_5Neu5Ac_1$	C-S	0.929	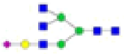
70	1769.635	$Hex_4HexNAc_4Neu5Ac_1$	C-S	0.925	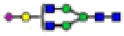
71	2524.931	$Hex_4HexNAc_7Fuc_1Neu5Ac_1$	C-FS	0.923	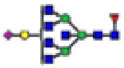

72	2808.010	$Hex_7HexNAc_6Fuc_1Neu5Ac_1$	C-FS	0.921	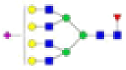
73	2866.052	$Hex_7HexNAc_7Fuc_2$	C-F	0.920	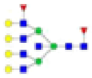
74	1259.465	$Hex_3HexNAc_3Fuc_1$	C-F	0.907	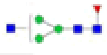
75	2880.031	$Hex_6HexNAc_5Fuc_2Neu5Ac_2$	C/H-FS	0.906	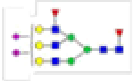
76	1599.566	$Hex_6HexNAc_3$	H	0.887	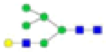
77	2833.042	$Hex_5HexNAc_7Fuc_2Neu5Ac_1$	C-FS	0.886	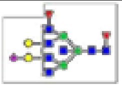
78	2093.740	$Hex_6HexNAc_4Neu5Ac_1$	H-S	0.870	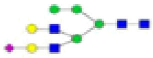
79	2775.000	$Hex_5HexNAc_6Fuc_1Neu5Ac_2$	C-FS	0.853	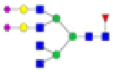
80	2249.830	$Hex_5HexNAc_7$	C	0.851	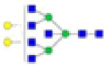
81	2135.787	$Hex_5HexNAc_5Fuc_2$	C/H-F	0.850	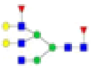
82	2587.915	$Hex_6HexNAc_5Neu5Ac_2$	C/H-S	0.843	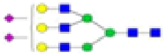
83	2790.995	$Hex_6HexNAc_6Neu5Ac_2$	C-S	0.809	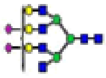
84	2222.783	$Hex_5HexNAc_4Neu5Ac_2$	C-S	0.801	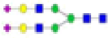
85	2176.814	$Hex_4HexNAc_6Fuc_2$	C-F	0.795	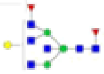
86	2425.862	$Hex_5HexNAc_5Neu5Ac_2$	C-S	0.789	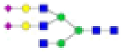
87	3026.089	$Hex_6HexNAc_5Fuc_3Neu5Ac_2$	C/H-FS	0.784	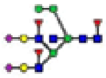
88	2208.804	$Hex_6HexNAc_6$	C	0.765	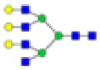
89	2645.957	$Hex_6HexNAc_6Fuc_1Neu5Ac_1$	C-FS	0.760	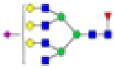
90	2525.951	$Hex_4HexNAc_7Fuc_3$	C-F	0.757	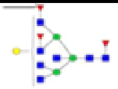
91	1380.491	$GlcNAc_2Man_5Fuc_1$	HM-F	0.756	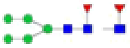

92	1421.518	$Hex_4HexNAc_3Fuc_1$	C/H-F	0.749	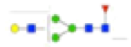
93	2541.946	$Hex_5HexNAc_7Fuc_2$	C-F	0.748	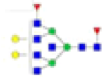
94	2540.926	$Hex_5HexNAc_7Neu5Ac_1$	C-S	0.717	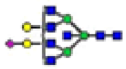
95	2296.820	$Hex_6HexNAc_5Neu5Ac_1$	C/H-S	0.710	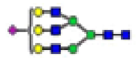
96	2044.697	$GlcNAc_2Man_{10}$	HM	0.697	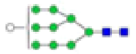
97	3229.168	$Hex_6HexNAc_6Fuc_3Neu5Ac_2$	C-FS	0.694	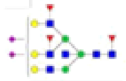
98	1770.655	$Hex_4HexNAc_4Fuc_2$	C/H-F	0.690	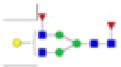
99	2516.914	$Hex_7HexNAc_6Fuc_1$	C-F	0.689	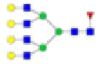
100	2588.936	$Hex_6HexNAc_5Fuc_2Neu5Ac_1$	C/H-FS	0.684	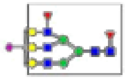
101	2411.883	$Hex_6HexNAc_7$	C	0.680	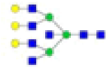
102	2614.988	$Hex_4HexNAc_6Fuc_5$	C-F	0.678	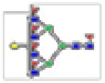
103	2442.878	$Hex_6HexNAc_5Fuc_1Neu5Ac_1$	C/H-FS	0.668	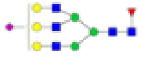
104	2849.036	$Hex_6HexNAc_7Fuc_1Neu5Ac_1$	C-FS	0.666	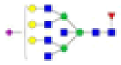
105	3083.110	$Hex_6HexNAc_6Fuc_2Neu5Ac_2$	C-FS	0.664	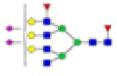
106	2467.909	$Hex_4HexNAc_6Fuc_2Neu5Ac_1$	C-FS	0.663	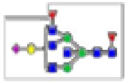
107	2703.999	$Hex_6HexNAc_7Fuc_2$	C-F	0.660	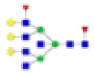
108	2030.756	$Hex_4HexNAc_6Fuc_1$	C-F	0.634	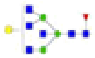
109	2978.079	$Hex_5HexNAc_7Fuc_1Neu5Ac_2$	C-FS	0.617	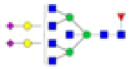
110	2646.977	$Hex_6HexNAc_6Fuc_3$	C-F	0.602	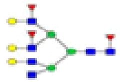
111	3140.132	$Hex_6HexNAc_7Fuc_1Neu5Ac_2$	C-FS	0.597	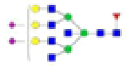

112	2557.941	$Hex_6HexNAc_7Fuc_1$	C-F	0.596	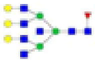
113	2077.745	$Hex_5HexNAc_4Fuc_1Neu5Ac_1$	C/H-FS	0.594	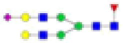
114	3011.089	$Hex_7HexNAc_7Fuc_1Neu5Ac_1$	C-FS	0.592	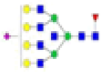
115	2426.883	$Hex_5HexNAc_5Fuc_2Neu5Ac_1$	C/H-FS	0.592	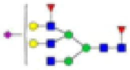
116	1874.666	$Hex_5HexNAc_3Fuc_1Neu5Ac_1$	H-FS	0.584	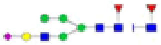
117	2354.862	$Hex_6HexNAc_6Fuc_1$	C-F	0.577	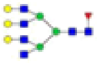
118	2233.835	$Hex_4HexNAc_7Fuc_1$	C-F	0.570	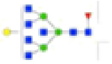
119	1583.571	$Hex_5HexNAc_3Fuc_1$	H-F	0.566	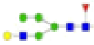
120	2571.920	$Hex_5HexNAc_5Fuc_1Neu5Ac_2$	C-FS	0.566	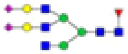
121	1948.703	$Hex_6HexNAc_4Fuc_1$	H-F	0.564	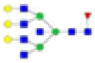
122	2719.994	$Hex_7HexNAc_7Fuc_1$	C-F	0.563	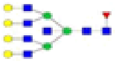
123	2192.809	$Hex_5HexNAc_6Fuc_1$	C-F	0.561	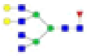
124	1811.682	$Hex_3HexNAc_5Fuc_2$	C-F	0.553	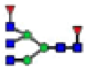
125	2395.888	$Hex_5HexNAc_7Fuc_1$	C-F	0.547	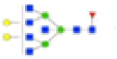
126	1542.544	$GlcNAc_2Man_6Fuc_1$	HM-F	0.547	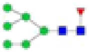
127	1786.650	$Hex_5HexNAc_4Fuc_1$	C/H-F	0.544	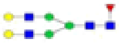
128	2281.845	$Hex_5HexNAc_5Fuc_3$	C/H-F	0.542	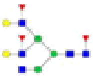
129	1964.698	$Hex_7HexNAc_4$	H	0.528	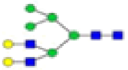
130	1608.602	$Hex_3HexNAc_4Fuc_2$	C-F	0.511	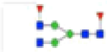
131	2662.972	$Hex_7HexNAc_6Fuc_2$	C-F	0.492	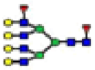

132	1728.608	$Hex_5HexNAc_3Neu5Ac_1$	H-S	0.489	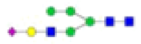
133	2402.871	$Hex_7HexNAc_4Fuc_3$	H-F	0.488	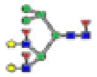
134	1566.555	$Hex_4HexNAc_3Neu5Ac_1$	C-S	0.484	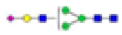
135	1890.661	$Hex_6HexNAc_3Neu5Ac_1$	H-S	0.482	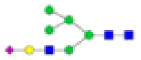
136	2573.936	$Hex_7HexNAc_7$	C	0.480	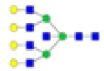
137	2151.782	$Hex_6HexNAc_5Fuc_1$	C/H-F	0.479	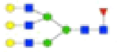
138	2005.724	$Hex_6HexNAc_5$	C/H	0.467	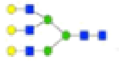
139	2660.957	$Hex_5HexNAc_4Fuc_3Neu5Ac_2$	C-FS	0.465	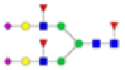
140	2207.808	$Hex_4HexNAc_4Fuc_3Neu5Ac_1$	C-FS	0.459	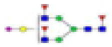
141	2717.978	$Hex_5HexNAc_5Fuc_2Neu5Ac_2$	C-FS	0.445	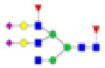
142	1056.386	$GlcNAc_2Man_3Fuc_1$	HM-F	0.441	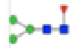
143	2338.867	$Hex_5HexNAc_6Fuc_2$	C-F	0.440	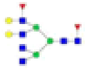
144	2175.794	$Hex_4HexNAc_6Neu5Ac_1$	C-S	0.431	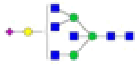
145	2832.021	$Hex_5HexNAc_7Neu5Ac_2$	C-S	0.424	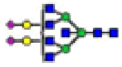
146	2264.830	$Hex_4HexNAc_5Fuc_2Neu5Ac_1$	C-FS	0.421	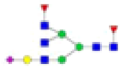
147	2443.898	$Hex_6HexNAc_5Fuc_3$	C/H-F	0.421	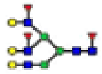
148	2823.009	$Hex_6HexNAc_4Fuc_3Neu5Ac_2$	H-FS	0.414	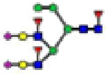
149	2036.719	$Hex_6HexNAc_3Fuc_1Neu5Ac_1$	H-FS	0.413	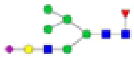
150	2572.941	$Hex_5HexNAc_5Fuc_3Neu5Ac_1$	C/H-FS	0.402	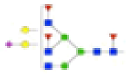
151	1931.688	$Hex_5HexNAc_4Neu5Ac_1$	C/H-S	0.384	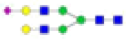

231

152	2500.920	$Hex_6HexNAc_6Fuc_2$	C-F	0.374	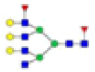
153	2368.841	$Hex_5HexNAc_4Fuc_1Neu5Ac_2$	C-FS	0.370	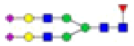
154	1218.438	$GlcNAc_2Man_4Fuc_1$	HM-F	0.352	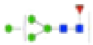
155	2239.798	$Hex_6HexNAc_4Fuc_1Neu5Ac_1$	H-FS	0.296	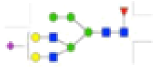
156	2255.793	$Hex_7HexNAc_4Neu5Ac_1$	H-S	0.285	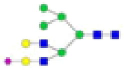
157	2028.702	$GlcNAc_2Man_9Fuc_1$	HM-F	0.283	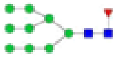
158	1704.597	$GlcNAc_2Man_7Fuc_1$	HM-F	0.276	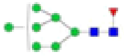
159	3025.068	$Hex_6HexNAc_5Fuc_1Neu5Ac_3$	C-FS	0.274	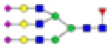
160	2401.851	$Hex_7HexNAc_4Fuc_1Neu5Ac_1$	H-FS	0.267	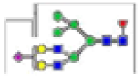
161	3158.168	$Hex_7HexNAc_7Fuc_4$	C-F	0.235	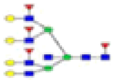
162	2378.873	$Hex_4HexNAc_7Neu5Ac_1$	C-S	0.228	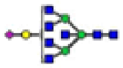
163	2661.952	$Hex_7HexNAc_6Neu5Ac_1$	C-S	0.223	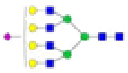
164	1932.708	$Hex_5HexNAc_4Fuc_2$	C/H-F	0.207	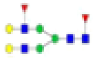
165	2954.068	$Hex_7HexNAc_6Fuc_2Neu5Ac_1$	C-FS	0.196	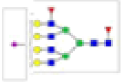
166	3448.243	$Hex_7HexNAc_7Fuc_2Neu5Ac_2$	C-FS	0.184	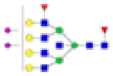
167	3228.148	$Hex_6HexNAc_6Fuc_1Neu5Ac_3$	C-FS	0.182	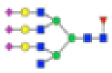
168	1916.713	$Hex_4HexNAc_4Fuc_3$	C/H-F	0.171	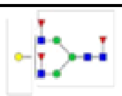
169	2777.040	$Hex_5HexNAc_6Fuc_5$	C-F	0.169	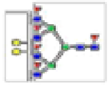
170	2410.888	$Hex_4HexNAc_5Fuc_3Neu5Ac_1$	C-FS	0.166	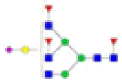
171	2337.846	$Hex_5HexNAc_6Neu5Ac_1$	C-S	0.161	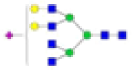

232

172	2955.088	$Hex_7HexNAc_6Fuc_4$	C-F	0.147	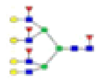
173	2509.956	$Hex_3HexNAc_7Fuc_4$	C-F	0.138	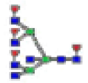
174	3593.280	$Hex_7HexNAc_7Fuc_1Neu5Ac_3$	C-FS	0.133	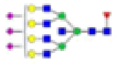
175	1891.681	$Hex_6HexNAc_3Fuc_2$	H-F	0.126	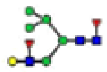
176	3084.131	$Hex_6HexNAc_6Fuc_4Neu5Ac_1$	C-FS	0.082	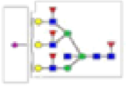
177	2879.011	$Hex_6HexNAc_5Neu5Ac_3$	C-S	0.067	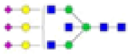
178	3375.226	$Hex_6HexNAc_6Fuc_4Neu5Ac_2$	C-FS	0.041	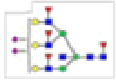
179	2110.756	$Hex_7HexNAc_4Fuc_1$	H-F	0.000	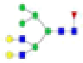
180	1405.523	$Hex_3HexNAc_3Fuc_2$	C-F	0.000	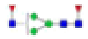
181	2322.872	$Hex_4HexNAc_6Fuc_3$	C-F	0.000	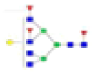
182	2896.026	$Hex_7HexNAc_5Fuc_1Neu5Ac_2$	H-FS	0.000	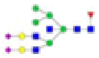
183	3431.227	$Hex_6HexNAc_7Fuc_1Neu5Ac_3$	C-FS	0.000	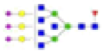
184	3390.201	$Hex_7HexNAc_6Fuc_1Neu5Ac_3$	C-FS	0.000	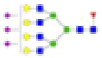
185	3286.190	$Hex_6HexNAc_7Fuc_2Neu5Ac_2$	C-FS	0.000	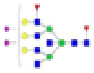
186	3374.206	$Hex_6HexNAc_6Fuc_2Neu5Ac_3$	C-FS	0.000	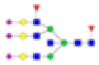
187	2979.099	$Hex_5HexNAc_7Fuc_3Neu5Ac_1$	C-FS	0.000	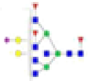
188	2760.025	$Hex_4HexNAc_6Fuc_4Neu5Ac_1$	C-FS	0.000	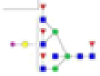
189	2499.899	$Hex_6HexNAc_6Neu5Ac_1$	C-S	0.000	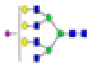
190	3156.127	$Hex_7HexNAc_7Neu5Ac_2$	C-S	0.000	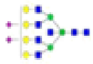
191	3082.090	$Hex_6HexNAc_6Neu5Ac_3$	C-S	0.000	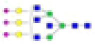

192	2850.057	$Hex_6HexNAc_7Fuc_3$	C-F	Undefined	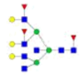
193	3012.110	$Hex_7HexNAc_7Fuc_3$	C-F	Undefined	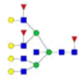
194	2385.856	$Hex_6HexNAc_4Fuc_2Neu5Ac_1$	H-FS	Undefined	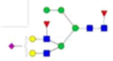
195	3141.152	$Hex_6HexNAc_7Fuc_3Neu5Ac_1$	C-FS	Undefined	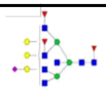
196	2693.967	$Hex_7HexNAc_4Fuc_3Neu5Ac_1$	H-FS	Undefined	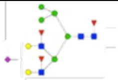
197	3449.263	$Hex_7HexNAc_7Fuc_4Neu5Ac_1$	C-FS	Undefined	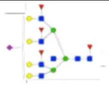
198	3521.284	$Hex_6HexNAc_6Fuc_5Neu5Ac_2$	C-FS	Undefined	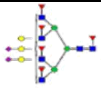

*The observed mass from analysis was used to match the theoretical mass in the retrosynthetic database[1].

Supplementary Table S3: Differentially expressed genes following *Salmonella* LT2 challenge. Caco-2 cells treated with *Salmonella* LT2 for 60 minutes displayed a significant induction directly involved in glycan metabolism. Gene induction reported as Log_2 ratio (*Salmonella* treated/not treated Caco-2) ($p \leq 0.05$, FDR 0.01).

**Symbol**	**Entrez Gene Name**	**Log_2 Ratio**	**Cellular Location**
ALG3	ALG3, alpha-1,3- mannosyltransferase	1.004	Cytoplasm
ALG9	ALG9, alpha-1,2-mannosyltransferase	0.481	Cytoplasm
B3GALNT2	beta-1,3-*N*-acetylgalactosaminyltransferase 2	0.718	Cytoplasm
B3GALT5	UDP-Gal:betaGlcNAc beta 1,3-galactosyltransferase, polypeptide 5	0.098	Cytoplasm
B3GAT3	beta-1,3-glucuronyltransferase 3 (glucuronosyltransferase I)	0.576	Cytoplasm
B3GNT1	UDP-GlcNAc:betaGal beta-1,3-*N*-acetylglucosaminyltransferase 1	0.820	Cytoplasm
B3GNT2	UDP-GlcNAc:betaGal beta-1,3-*N*-acetylglucosaminyltransferase 2	0.159	Cytoplasm
B3GNT3	UDP-GlcNAc:betaGal beta-1,3-*N*-acetylglucosaminyltransferase 3	0.293	Cytoplasm
B3GNT4	UDP-GlcNAc:betaGal beta-1,3-*N*-acetylglucosaminyltransferase 4	0.366	Plasma Membrane
B3GNT7	UDP-GlcNAc:betaGal beta-1,3-*N*-acetylglucosaminyltransferase 7	0.726	Cytoplasm
B3GNTL1	UDP-GlcNAc:betaGal beta-1,3-*N*-acetylglucosaminyltransferase-like 1	0.811	Other
B4GALNT4	beta-1,4-*N*-acetyl-galactosaminyl transferase 4	0.189	Extracellular Space
B4GALT1	UDP-Gal:betaGlcNAc beta 1,4-galactosyltransferase, polypeptide 1	1.371	Cytoplasm
B4GALT3	UDP-Gal:betaGlcNAc beta 1,4-galactosyltransferase, polypeptide 3	0.799	Cytoplasm
B4GALT7	xylosylprotein beta 1,4-galactosyltransferase, polypeptide 7	0.439	Cytoplasm
DPAGT1	dolichyl-phosphate (UDP-*N*-acetylglucosamine) *N*-acetylglucosaminephosphotransferase 1 (GlcNAc-1-P transferase)	1.063	Cytoplasm
EDEM1	ER degradation enhancer, mannosidase alpha-like 1	0.319	Cytoplasm
EDEM2	ER degradation enhancer, mannosidase alpha-like 2	0.532	Cytoplasm
FUCA2	fucosidase, alpha-L- 2, plasma	0.108	Extracellular Space
FUT1	fucosyltransferase 1 (galactoside 2-alpha-L-fucosyltransferase, H blood group)	0.522	Cytoplasm
FUT11	fucosyltransferase 11 (alpha (1,3) fucosyltransferase)	0.489	Cytoplasm

FUT2	fucosyltransferase 2 (secretor status included)	1.004	Cytoplasm
FUT4	fucosyltransferase 4 (alpha (1,3) fucosyltransferase, myeloid-specific)	0.250	Cytoplasm
FUT5	fucosyltransferase 5 (alpha (1,3) fucosyltransferase)	0.166	Cytoplasm
FUT6	fucosyltransferase 6 (alpha (1,3) fucosyltransferase)	0.345	Cytoplasm
FUT7	fucosyltransferase 7 (alpha (1,3) fucosyltransferase)	0.310	Cytoplasm
FUT9	fucosyltransferase 9 (alpha (1,3) fucosyltransferase)	0.063	Cytoplasm
POFUT1	*O*-fucosyltransferase 1 - protein	0.659	Cytoplasm
POFUT2	*O*-fucosyltransferase 2 - protein	0.622	Cytoplasm
GANAB	glucosidase, alpha; neutral AB	0.829	Cytoplasm
GLB1	galactosidase, beta 1	0.672	Cytoplasm
GLB1L2	galactosidase, beta 1-like 2	0.463	Other
MAN1A2	mannosidase, alpha, class 1A, member 2	0.256	Cytoplasm
MAN1B1	mannosidase, alpha, class 1B, member 1	0.583	Cytoplasm
MAN1C1	mannosidase, alpha, class 1C, member 1	0.391	Cytoplasm
MAN2A2	mannosidase, alpha, class 2A, member 2	0.342	Cytoplasm
MAN2B1	mannosidase, alpha, class 2B, member 1	0.863	Cytoplasm
MAN2B2	mannosidase, alpha, class 2B, member 2	0.619	Cytoplasm
MAN2C1	mannosidase, alpha, class 2C, member 1	0.624	Cytoplasm
MANBAL	mannosidase, beta A, lysosomal-like	0.577	Other
MANEAL	mannosidase, endo-alpha-like	0.235	Other
MGAT1	mannosyl (alpha-1,3-)-glycoprotein beta-1,2-*N*-acetylglucosaminyltransferase	0.559	Cytoplasm
MGAT3	mannosyl (beta-1,4-)-glycoprotein beta-1,4-*N*-acetylglucosaminyltransferase	0.712	Cytoplasm
MGAT4B	mannosyl (alpha-1,3-)-glycoprotein beta-1,4-*N*-acetylglucosaminyltransferase, isozyme B	0.810	Cytoplasm
MGAT5	mannosyl (alpha-1,6-)-glycoprotein beta-1,6-*N*-acetyl-glucosaminyltransferase	2.156	Cytoplasm
MOGS	mannosyl-oligosaccharide glucosidase	0.684	Cytoplasm
MOGAT3	monoacylglycerol *O*-acyltransferase 3	0.890	Other
NAGA	*N*-acetylgalactosaminidase, alpha-	0.942	Cytoplasm
NAGK	*N*-acetylglucosamine kinase	0.519	Cytoplasm
NAGLU	*N*-acetylglucosaminidase, alpha	0.479	Cytoplasm
NAGPA	*N*-acetylglucosamine-1-phosphodiester alpha-*N*-acetylglucosaminidase	0.380	Cytoplasm
NAGS	*N*-acetylglutamate synthase	0.614	Cytoplasm
NANS	*N*-acetylneuraminic acid synthase	0.152	Cytoplasm
NEU1	sialidase 1 (lysosomal sialidase)	0.789	Cytoplasm, lysosome
NEU3	sialidase 3 (membrane sialidase)	0.214	Plasma Membrane
SIGLEC11	sialic acid binding Ig-like lectin 11	0.483	Plasma Membrane
SIGLEC5	sialic acid binding Ig-like lectin 5	0.419	Plasma Membrane
SIGLEC6	sialic acid binding Ig-like lectin 6	0.545	Extracellular

			Space
ST3GAL1	ST3 beta-galactoside alpha-2,3-sialyltransferase 1	0.900	Cytoplasm
ST3GAL2	ST3 beta-galactoside alpha-2,3-sialyltransferase 2	0.742	Cytoplasm
ST3GAL4	ST3 beta-galactoside alpha-2,3-sialyltransferase 4	0.253	Cytoplasm
ST6GAL1	ST6 beta-galactosamide alpha-2,6-sialyltranferase 1	0.429	Cytoplasm
ST6GAL2	ST6 beta-galactosamide alpha-2,6-sialyltranferase 2	0.288	Cytoplasm
ST6GALNAC1	ST6 (alpha-*N*-acetyl-neuraminyl-2,3-beta-galactosyl-1,3)-*N*-acetylgalactosaminide alpha-2,6-sialyltransferase 1	0.482	Cytoplasm
ST6GALNAC2	ST6 (alpha-*N*-acetyl-neuraminyl-2,3-beta-galactosyl-1,3)-*N*-acetylgalactosaminide alpha-2,6-sialyltransferase 2	0.327	Cytoplasm
NGLY1	*N*-glycanase 1	0.957	Cytoplasm
STT3A	STT3A, subunit of the oligosaccharyltransferase complex (catalytic)	0.162	Plasma Membrane
STT3B	STT3B, subunit of the oligosaccharyltransferase complex (catalytic)	0.856	Cytoplasm

Supplementary Table S4. Human sialidase and sialyltransferase expression during infection with *Salmonella* LT2. Data adapted from IPA, Crespo et al.[2] and this study.

**Symbol**	**Name**	**Cellular Location**	**Preferred substrates**	**Glycan specificity**	**Function**	**Regulation**
Neu1	Neuraminidase 1	Lysozome	Siaα2,3<br>Siaα2,6	Glycoproteins<br>Glycolipids	Cleaves terminal sialic acid residues from substrates such as glycoproteins and glycolipids	Induced
Neu2	Neuraminidase 2	Cytosol	Siaα2,3<br>Siaα2,6	Glycoproteins<br>Glycolipids	Cleaves terminal sialic acid residues from substrates such as glycoproteins and glycolipids	Not regulated
Neu3	Neuraminidase 3	Cell membrane	Siaα2,3<br>Siaα2,6	Gangliosides<br>Lactosylceramide	Preferentially hydrolyzes gangliosides with alpha-2,3 or alpha-2,6 linkages	Induced
Neu4	Neuraminidase 4	Inner membrane	Siaα2,3<br>Siaα2,6	Glycoproteins<br>Glycolipids<br>Gangliosides	Remove terminal sialic acid residues from various sialo derivatives	Not regulated
**ST3GAL family - catalyzes the addition of sialic acids to a terminal galactose of glycans in an α-2,3 linkage**						
ST3GAL-1	ST3 β-galactoside α-2,3-sialyltransferase 1	Cell membrane<br>Cytoplasm	Galβ1,3GalNAc	*O*-glycan	Catalyzes the transfer of sialic acid from CMP-sialic acid to galactose-containing substrates	Induced
ST3GAL-2	ST3 β-galactoside α-2,3-sialyltransferase 2	Cell membrane<br>Cytoplasm	Galβ1,3GalNAc	*O*-glycan	Catalyzes the transfer of sialic acid from CMP-sialic acid to	Induced

					galactose-containing substrates	
ST3GAL-3	ST3 β-galactoside α-2,3-sialyltransferase 3	Cell membrane Cytoplasm	Galβ1,3(4)GlcNAc	*O*-glycan, *N*-glycan	Catalyzes the transfer of sialic acid from CMP-sialic acid to galactose-containing substrates	Not regulated
ST3GAL-4	ST3 β-galactoside α-2,3-sialyltransferase 4	Cell membrane Cytoplasm	Galβ1,4(3)GlcNAc	*N*-glycan, *O*-glycan	Catalyzes the transfer of sialic acid from CMP-sialic acid to galactose-containing substrates	Induced
ST3GAL-5	ST3 β-galactoside α-2,3-sialyltransferase 5	Cell membrane Cytoplasm	Galβ1,4Glc-ceramide	Glycolipid	Catalyzes the transfer of sialic acid from CMP-sialic acid to galactose-containing substrates	Not regulated
ST3GAL-6	ST3 β-galactoside α-2,3-sialyltransferase 6	Cell membrane Cytoplasm	Galβ1,4GlcNAc	*N*-glycan	Catalyzes the transfer of sialic acid from CMP-sialic acid to galactose-containing substrates	Not regulated
**ST6GAL family - transfers α-2,6 linking sialic acids to a terminal galactose residues of *N*-glycans**						
ST6GAL-1	ST6 β-galactoside α-2,6-sialyltransferase 1	Cell membrane Cytoplasm	Galβ1,4GlcNAc	*N*-glycan	Catalyzes the transfer of sialic acid from CMP-sialic acid to galactose-containing substrates. Considered to be an inflammatory marker.	Induced
ST6GAL-2	ST6 β-galactoside α-2,6-sialyltransferase 2	Cell membrane Cytoplasm	Galβ1,4GlcNAc	*N*-glycan	Catalyzes the transfer of sialic acid from CMP-sialic acid to galactose-containing	Induced

					substrates	
**ST6GALNAc family - adds sialic acids to terminal *N*-acetylgalactosamine residues of glycoproteins and glycolipids in an α-2,6 linkage**						
ST6GALNAc-1	ST6 (α-*N*-acetyl-neuraminyl-2,3-β-galactosyl-1,3)-*N*-acetylgalactosaminide α-2,6-sialyltransferase 1	Cell membrane Cytoplasm	GalNAcα1 Galβ1,3GalNAcα1	*O*-glycan	Transfers a sialic acid, *N*-acetylneuraminic acid (NeuAc), in an α-2,6 linkage to O-linked GalNAc residues	Induced
ST6GALNAc-2	ST6 (α-*N*-acetyl-neuraminyl-2,3-β-galactosyl-1,3)-*N*-acetylgalactosaminide α-2,6-sialyltransferase 2	Cell membrane Cytoplasm	Galβ1,3GalNAcα1	*O*-glycan	Add sialic acids to the nonreducing ends of glycoconjugates. At the cell surface, these modifications have roles in cell-cell and cell-substrate interactions, bacterial adhesion, and protein targeting	Induced
ST6GALNAc-3	ST6 (α-*N*-acetyl-neuraminyl-2,3-β-galactosyl-1,3)-*N*-acetylgalactosaminide α-2,6-sialyltransferase 3	Cell membrane Cytoplasm	Siaα2,3Galβ1,3GalNAc	*O*-glycan	Transfer sialic acids from CMP-sialic acid to terminal positions of carbohydrate groups in glycoproteins and glycolipids	Not regulated
ST6GALNAc-4	ST6 (α-*N*-acetyl-neuraminyl-2,3-β-galactosyl-1,3)-*N*-acetylgalactosaminide α-2,6-sialyltransferase 4	Cell membrane Cytoplasm	Siaα2,3Galβ1,3GalNAc	*O*-glycan	Catalyzes the transfer of sialic acid from CMP-sialic acid to galactose-containing substrates. The encoded protein prefers glycoproteins rather than glycolipids as substrates and shows	Not regulated

					restricted substrate specificity, utilizing only the trisaccharide sequence Neu5Ac-α-2,3-Gal-β-1,3-GalNAc. In addition, it is involved in the synthesis of ganglioside GD1A from GM1B.	
ST6GALNAc-5	ST6 (α-*N*-acetyl-neuraminyl-2,3-β-galactosyl-1,3)-*N*-acetylgalactosaminide α-2,6-sialyltransferase 5	Cell membrane Cytoplasm	GM1b	Glycolipid	Modify proteins and ceramides on the cell surface to alter cell-cell or cell-extracellular matrix interactions	Not regulated
ST6GALNAc-6	ST6 (α-*N*-acetyl-neuraminyl-2,3-β-galactosyl-1,3)-*N*-acetylgalactosaminide α-2,6-sialyltransferase 6	Cell membrane Cytoplasm	All α-series gangliosides	Glycolipid	Modify proteins and ceramides on the cell surface to alter cell-cell or cell-extracellular matrix interactions	Not regulated
**ST8SIA family - promotes the linkage to another sialic acid residue in *N*- or *O*-glycans in an α-2,8 linkage (expressed in neural cells)**						
ST8SIA-1	ST8 α-*N*-acetyl-neuraminide α-2,8-sialyltransferase 1	Cell membrane Cytoplasm	Siaα2,3Galβ1,4Glc-ceramide	Glycolipid	Catalyzes the transfer of sialic acid from CMP-sialic acid to GM3 to produce gangliosides GD3 and GT3	Not regulated
ST8SIA-2	ST8 α-*N*-acetyl-neuraminide α-2,8-sialyltransferase 2	Cell membrane Cytoplasm	Siaα2,3Galβ1,4GlcNAc	*N*-glycan	Catalyze the transfer of sialic acid from CMP-sialic acid to *N*-linked oligosaccharides and glycoproteins. The encoded protein may be	Not regulated

					found in the Golgi apparatus and may be involved in the production of polysialic acid, a modulator of the adhesive properties of neural cell adhesion molecule (NCAM1)	
ST8SIA-3	ST8 α-*N*-acetyl-neuraminide α-2,8-sialyltransferase 3	Cell membrane Cytoplasm	Siaα2,3Galβ1,4GlcNAc	*N*-glycan	Forms sialyl-α-2,8-sialyl-R linkages at the nonreducing termini of glycoconjugates	Not regulated
ST8SIA-4	ST8 α-*N*-acetyl-neuraminide α-2,8-sialyltransferase 4	Cell membrane Cytoplasm	(Sialα2,8)nSiaα2,3Galβ1-R	*N*-glycan	Catalyzes the polycondensation of α-2,8-linked sialic acid required for the synthesis of polysialic acid, a modulator of the adhesive properties of neural cell adhesion molecule (NCAM1)	Not regulated
ST8SIA-5	ST8 α-*N*-acetyl-neuraminide α-2,8-sialyltransferase 5	Cell membrane Cytoplasm	GM1b, GT1b, GD1a, GD3	Glycolipid	Involved in the synthesis of gangliosides GD1c, GT1a, GQ1b, and GT3 from GD1a, GT1b, GM1b, and GD3, respectively	Not regulated
ST8SIA-6	ST8 α-*N*-acetyl-neuraminide α-2,8-sialyltransferase 6	Cell membrane Cytoplasm	Siaα2,3(6)Gal	Sialic acid on *O*-glycan	Synthesize sialylglycoconjugates	Not regulated

Supplementary Table S5: Strains and associated PCR primers used for deletion mutant construction. The deletion mutants that did not change infection were not included in the genomic analysis.

**Gene::Strain Name**	**Primers used for generation**	**Notes**
Salmonella LT2	N/A	Wild-type strain Accession AE006468
ΔSTM2896::Cm LT2	5′-GTCGTACTATTGAAAAGCTGTCTTAATTTAATATTAACAGGATACCTATAGTGTAGGCTGGAGCTGCTTC-3′<br>5′-TAATTCAGCGATATCCAAATGTTGCATAGATCTTTTCCTTAATTAAGCCCATGGGAATTAGCCATGGTCC-3′	ΔinvA, strain is chloramphenicol (Cm) resistant Accession
ΔSTM4298::Cm LT2	5'-ACCGGGAACGCGTGCCACCATGAAGGTGTTCACTACTGTTCTGGAGAGTCGTGTAGGCTGGAGCTGCTTC-3'<br>5'-TCTGTTAGTAGAGGTTAAACGCCGGATCTGCCGTCCAGCGCGGCTGGAGAATGGGAATTAGCCATGGTCC-3'	Strain is Cm resistant Accession
ΔSTM1252::Cm LT2	5'-TTCATACGTAAAAAAGCTGATGCTTTTTACCTTATTTAATCGAGGCAGGTGTGTAGGCTGGAGCTGCTTC-3'<br>5'-CCGGGCAAGGCATTATTATTCCAGCGCTATCGGTTTCGACTGATAGCGCCATGGGAATTAGCCATGGTCC-3'	Strain is Cm resistant Accession
ΔSTM0928::Cm LT2	5′-ACTCTTCTGTGCTACTCGTCACATAAACAGAAATAACGGTAATCATATGTGTGTAGGCTGGAGCTGCTTC-3′<br>5′-TAGAGACTGCTGCTATATGTTATATAGCAGCAATGGCTATTTTTTGATGTATGGGAATTAGCCATGGTCC-3′	Strain is Cm resistant Accession
ΔSTM3664::Cm LT2	5′-CGCCTAAAAACCAGGCAAGCTGATGAATATTGCCCACAAAGGATAGCGTGGTGTAGGCTGGAGCTGCTTC-3′<br>5′-TTTTGTTTTGAAGGGGCTACCGGTACGCGAGGAGACCGGTAGCGCCACGAATGGGAATTAGCCATGGTCC-3′	Strain is Cm resistant Accession
ΔSTM3537::Cm LT2	5'-AACCTGACGTTACCGCCGTTGGCGACGATCTGGCTGATGCGGGAGGGGGAGTGTAGGCTGGAGCTGCTTC-3'<br>5'-CAACATTACACGATCGTTCTTCTCTAAACTCACCATGATTAACTCCTTTTATGGGAATTAGCCATGGTCC-3'	Strain is Cm resistant Accession
ΔSTM3338::Cm LT2	5'-GCGGAGCCGCCATTCGGCATAACGACAACAAAAATCGTCGGGAGAGTAAAGTGTAGGCTGGAGCTGCTTC-3'<br>5'-ACATACTACTCCCTTACAAATATTGCCCGGTTGAAAACCGGGCAAGCGGAATGGGAATTAGCCATGGTCC-3'	Strain is Cm resistant
ΔSTM3662::Cm LT2	5'-GTTTTGATAGTATCCATGAGTCCAGGTTCACTTTTGCAGGATATATCCTCGTGTAGGCTGGAGCTGCTTC-3'<br>5'-CGGATGGCGCTACGCTATCCGGCTTACAAGGCATCTGTAGGCTGGATAAAATGGGAATTAGCCATGGTCC-3'	Strain is Cm resistant
ΔSTM3663::Cm LT2	5'-TCGTGAAAAATAAAAGGTGTTGGAATGTATCAATCCGACCAGGAGACCTAGTGTAGGCTGGAGCTGCTTC-3'<br>5'-CTACAGATGCCTTGTAAGCCGGATAGCGTAGCGCCATCCGGCACACAGAAATGGGAATTAGCCATGGTCC-3'	Strain is Cm resistant

Supplementary Methods

Cell Culture

Colonic epithelial cells (Caco-2; ATCC HTB-37) were obtained from American Type Culture Collection (Manassas, VA) and grown as per the manufacturer's instructions. Briefly, cells were seeded to a density of 10^5 cells/cm^2 in 96-well plate using culture media comprising of DMEM/High Modified (Thermo Scientific, Rockford, IL), non-essential amino acids (Thermo Scientific, Rockford, IL), 10mM MOPS (Sigma, St. Louis, MO), 10 mM TES (Sigma, St. Louis, MO), 15 mM HEPES (Sigma, St. Louis, MO) and 2 mM NaH_2PO_4 (Sigma, St. Louis, MO). Additionally, 16.6% fetal bovine serum (FBS) (HyClone Laboratories, Logan, UT) was added to the cell culture media during maintenance and propagation between bacterial association assays. Cells were incubated at 37˚C with 5% CO_2 to allow differentiation before use in bacterial association assays[3,4].

Gene Deletion

Bacterial gene deletions were done as described by Datsenko and Wanner[5]. Deletion strains were generated using the lambda-red recombinase method as described. A mini-prep kit (Qiagen, Valencia, CA) was used to isolate plasmid pKD46 containing ampicillin resistance and λ Red recombinase genes, from *E. coli* BW25141 (CGSC 7634) and plasmid pKD3 carrying chloramphenicol resistance gene, from *E. coli* BW25141 (CGSC 7631). This plasmid pKD46 was electroporated into the parent (*Salmonella* WT). Transformants were selected on LB agar containing 100 µg/ml ampicillin (Sigma, St. Louis, MO). The parent strain containing pKD46 was grown in LB broth in the presence of 100 µg/ml ampicillin and 100 mM L-arabinose to induce λ Red recombinase production. Plasmid pKD3 (CmR) was used as a template to generate PCR products for deletion of each gene of interest. The primers used are shown in Supplementary Table S9. The purified PCR products were used for

transformation by electroporation (Bio-Rad Gene Pulser) of electrocompetent *Salmonella* WT with induced λ Red recombinase. The transformants were selected on LB agar with 10 μg/ml chloramphenicol. The gene deletion/interruption was confirmed using PCR for each junction site created by the insertion.

Bacterial Association Measurements

*Invaded Salmonella determination***.** To enumerate invaded bacteria, cells were incubated with 200 μl of 100 μg/ml gentamicin for 2 h at 37°C with 5% CO_2. Host cells incubated with cell culture media (without gentamicin) were used to enumerate total host associated bacteria. Cells were again washed once with 200 μl 1X PBS buffer and lysed with 50 μl of Warnex lysis buffer.

Quantitative bacterial analysis was done using qPCR with a CFX 96 Real Time System (BioRad, Hercules, CA). Reactions were done using SYBR Green Supermix (BioRad) as per manufacturer's instructions. 100 nM of forward (F) and reverse (R) PCR primers for EF-TU gene (F: 5'- ACG CGG TAT CAT CAA AGT GG - 3'; R: 5' - ATC GGG TGG ATC AGG GTA AC - 3') to quantify *Salmonella* WT and GAPDH gene (F: 5'- ACC ACA GTC CAT GCC ATC AC – 3'; R: 5'-TCC ACC ACC CTG TTG CTG TA -3') to quantify Caco-2 cells (Integrated DNA technologies, Coralville, IA) were used. The parameters for both primers was done using a denaturation step at 95°C for 5 min, followed by 40 cycles of denaturation, annealing, and extension at 95˚C for 15 s, 56˚C for 30 s, 72˚C for 30 s, respectively, and a final extension at 72°C for 1 min. The amplified product was verified using melt curve analysis from 50˚C to 95˚C with a transition rate of 0.2˚C/s.

Glycan Degradation

Cell membrane extraction. The cell pellets were suspended in 20 mM homogenized buffer containing 0.25 M sucrose, 20 mM Hepes-KOH (pH 7.4), and a protease inhibitor. Cells were lysed on ice using a sonicator (Qsonica, CT) and cell lysates were centrifuged at 2,000 x g for 10 min to remove the nuclear fraction and debris. The supernatant was collected and brought to 1 mL with homogenized buffer for ultracentrifugation at 60,000 rpm for 45 min at 4°C. The pellet was suspended in 0.2 M Na_2CO_3 (pH 11) and pelleted by ultracentrifugation to fragment the endoplasmic reticulum. Finally, to remove the cytoplasmic fraction, the pellet was washed with water followed by ultracentrifugation. The resulting membrane pellet was isolated and stored at -20°C until further processing.

Enzymatic Release of N-Glycans. Membrane pellets were suspended with 100 μL of 100 mM NH_4HCO_3 in 5 mM dithiothreitol and heated for 10 s at 100°C to thermally denature the proteins. To release the glycans, 2 μL of peptide N-glycosidase F (New England Biolabs, MA) were added to the samples and incubated at 60°C in a microwave reactor (CEM Corporation, NC) for 10 minutes at 20 watts. After addition of 400 μL of ice-cold ethanol, the samples were frozen for 1 hour at -80°C to precipitate residual deglycosylated proteins and centrifuged for 30 minutes at 15,000 rpm. The supernatant containing the *N*-glycans was collected and dried.

N-Glycan Enrichment. Released *N*-glycans were purified by solid-phased extraction containing a porous graphitized carbon (PGC) matrix. PGC cartridges were conditioned with nanopure water followed by 80% ACN in 0.05% TFA (v/v) solution and again with nanopure water. Glycan samples were loaded onto the cartridge and washed with nanopure water at a flow rate of 1 mL/min to remove salts and buffer. *N*-glycans were eluted with a solution of 40% ACN in 0.05% TFA (v/v) and dried.

Nano-LC-MS and LC-MS/MS Analysis. Samples were reconstituted in 15 μL of nanopure water and analyzed using an Agilent HPLC-Chip-QTOF MS (Agilent, CA). The microfluidic nanospray chip is connected to the microwell plate sampler and consists of an enrichment column and LC analytical

column packed with porous graphitized carbon. For each sample, 8 µL was injected onto the enrichment column and separated with a binary gradient: (A) 3% ACN and 0.1% formic acid (v/v) in water and (B) 90% ACN in 1% formic acid (v/v). The column was eluted at 0.3 µL/min for the nanopump and 4 µL/min for the capillary pump. The 32-min gradient was programmed with the following proportions and time points: 5% to 32.8% B from 0 min to 13.3 min; 32.8% to 35.9% B from 13.3 min to 16.5 min; 35.9% to 100% B from 16.5 to 21.5 at 0.8 µL/min to flush out non-glycan compounds; 100% to 0% from 21.5 to 32 min at 8 µL/min to re-equilibrate the column before the next injection. The drying gas, 2 L of filtered nitrogen gas and 2 L of filtered dry compressed air, was set to a temperature of 325˚C and a flow rate of 4 L/min. MS spectra were acquired in positive ionization mode at an acquisition time of 1.5 s per spectrum over a mass range of *m/z* 500-2000. Mass discrimination inaccuracies were corrected with reference masses of *m/z* 622.029, 922.010, 1221.991, and 1521.971.

Collision-induced dissociation (CID) was performed with nitrogen gas using a series of collision energies ($V_{collision}$) dependent on the *m/z* values of the *N*-glycans, based on the equation: $V_{collision}$ = slope (*m/z*) + offset, where the slope and offset were set at (1.8/100 Da) V and –2.4 V, respectively.

N-Glycan compositions were identified with an in-house retrosynthetic library according to accurate mass[1]. Signals above a signal-to-noise ratio of 5.0 were filtered and deconvoluted using MassHunter Qualitative Analysis B.03.01 (Agilent Technologies, CA). Deconvoluted masses were compared to theoretical masses using a mass tolerance of 20 ppm and a false discovery rate of 0.6%. Relative abundances were determined by integrating the peak areas for observed glycan masses and normalizing to the summed peak areas of all glycans detected.

Phylogenetic analysis of sialidases

Sialidase sequence searches in NCBI using Geneious were used to identify homologues of *nanH* (1900 amino acid sequences) and *CHPNeu* (1100 amino acid sequences). Amino acid sequences were aligned with Geneious Alignment with default parameters[6,7]. The multiple sequence analysis (MSA) were used to build phylogenetic trees using Geneious Tree Builder with Jukes-Cantor genetic distance model using Neighbor-Joining method with 1000 bootstrap replcations.

Supplementary References

1 Kronewitter, S. R. *et al.* The development of retrosynthetic glycan libraries to profile and classify the human serum N-linked glycome. *Proteomics* **9**, 2986-2994, doi:10.1002/pmic.200800760 (2009).

2 Crespo, H. J., Lau, J. T. & Videira, P. A. Dendritic cells: a spot on sialic Acid. *Frontiers in immunology* **4**, 491, doi:10.3389/fimmu.2013.00491 (2013).

3 Ferruzza, S., Rossi, C., Scarino, M. L. & Sambuy, Y. A protocol for differentiation of human intestinal Caco-2 cells in asymmetric serum-containing medium. *Toxicology in vitro : an international journal published in association with BIBRA* **26**, 1252-1255, doi:10.1016/j.tiv.2012.01.008 (2012).

4 Sambuy, Y. *et al.* The Caco-2 cell line as a model of the intestinal barrier: influence of cell and culture-related factors on Caco-2 cell functional characteristics. *Cell biology and toxicology* **21**, 1-26, doi:10.1007/s10565-005-0085-6 (2005).

5 Datsenko, K. A. & Wanner, B. L. One-step inactivation of chromosomal genes in Escherichia coli K-12 using PCR products. *Proceedings of the National Academy of Sciences of the United States of America* **97**, 6640-6645, doi:10.1073/pnas.120163297 (2000).

6 Edgar, R. C. MUSCLE: multiple sequence alignment with high accuracy and high throughput. *Nucleic acids research* **32**, 1792-1797, doi:10.1093/nar/gkh340 (2004).

7 Kearse, M. *et al.* Geneious Basic: an integrated and extendable desktop software platform for the organization and analysis of sequence data. *Bioinformatics* **28**, 1647-1649, doi:10.1093/bioinformatics/bts199 (2012).

5
